# *In planta* imaging of pyridine nucleotides using second‐generation fluorescent protein biosensors

**DOI:** 10.1111/tpj.16796

**Published:** 2024-05-18

**Authors:** Shey‐Li Lim, Jinhong Liu, Gilles Dupouy, Gaurav Singh, Stéphanie Baudrey, Lang Yang, Jia Yi Zhong, Marie‐Edith Chabouté, Boon Leong Lim

**Affiliations:** ^1^ School of Biological Sciences University of Hong Kong Hong Kong China; ^2^ Institut de Biologie Moléculaire des Plantes, CNRS Université de Strasbourg Strasbourg 67084 France; ^3^ Architecture et Réactivité de l'ARN Université de Strasbourg, CNRS UPR 9002 Strasbourg 67000 France; ^4^ HKU Shenzhen Institute of Research and Innovation Shenzhen China; ^5^ State Key Laboratory of Agrobiotechnology The Chinese University of Hong Kong Hong Kong China

**Keywords:** NADH, NADPH, plastid, pollen tube, root hair, ratiometric biosensor, redox, technical advance

## Abstract

Redox changes of pyridine nucleotides in cellular compartments are highly dynamic and their equilibria are under the influence of various reducing and oxidizing reactions. To obtain spatiotemporal data on pyridine nucleotides in living plant cells, typical biochemical approaches require cell destruction. To date, genetically encoded fluorescent biosensors are considered to be the best option to bridge the existing technology gap, as they provide a fast, accurate, and real‐time readout. However, the existing pyridine nucleotides genetically encoded fluorescent biosensors are either sensitive to pH change or slow in dissociation rate. Herein, we employed the biosensors which generate readouts that are pH stable for *in planta* measurement of NADH/NAD^+^ ratio and NADPH level. We generated transgenic Arabidopsis lines that express these biosensors in plastid stroma and cytosol of whole plants and pollen tubes under the control of CaMV 35S and LAT52 promoters, respectively. These transgenic biosensor lines allow us to monitor real‐time dynamic changes in NADH/NAD^+^ ratio and NADPH level in the plastids and cytosol of various plant tissues, including pollen tubes, root hairs, and mesophyll cells, using a variety of fluorescent instruments. We anticipate that these valuable transgenic lines may allow improvements in plant redox biology studies.

## Significant Statement

Monitoring dynamic changes in pyridine nucleotides (e.g., NADPH and NADH/NAD^+^ ratio) in plants at the subcellular level is a major obstacle in plant bioenergetics research. Genetically encoded fluorescent biosensors are considered to be the best option so far because they provide fast, accurate, and real‐time readouts. Here, we present a detailed study on the application of pyridine nucleotide biosensors, and we anticipate that these transgenic lines will be useful in plant redox biology studies.

## INTRODUCTION

The metabolic state of a plant cell is generally subjected to redox regulation, which is essential for the immediate adjustment of various metabolic pathways in different organelles to coordinate plant growth and development (Considine & Foyer, 2014). Pyridine nucleotides are important redox carriers in living plants and participate in many key bioenergetics processes, including, but not limited to linear electron flow, Calvin–Bassham–Benson cycle, and oxidative pentose phosphate pathway in the chloroplast (Heineke et al., 1991; Lim et al., 2020), glycolysis in the cytoplasm (Fernie et al., 2004), tricarboxylic acid cycle and electron transport chain in the mitochondria (Fernie et al., 2004), photorespiration, and nitrogen assimilation (Foyer et al., 2011; Lim et al., 2020). Moreover, the pyridine nucleotide pool can indirectly affect the growth status of plants. Owing to their indispensable roles in regulating cellular redox reactions, real‐time monitoring of subcellular redox states of these pyridine nucleotides within plant cells is crucial.

To date, a large number of studies have measured nicotinamide adenine dinucleotide (NADH), nicotinamide adenine dinucleotide phosphate (NADPH), and their oxidized forms (NAD^+^, NADP^+^) in plants by *in vitro* methods using bioluminescence (Kasimova et al., 2006; Latouche et al., 2000), high‐performance liquid chromatography, mass spectrometry (Agius et al., 2001), enzymatic assay, (Lowry & Passonneau, 1972), and radioactive methods (Brodelius & Vogel, 1985). However, these conventional methods require the extraction of NAD(H) and NADP(H) from the plant tissues before measurement. Destruction of plant tissues during a complex extraction process may distort the actual amount of pyridine nucleotides in plants. Furthermore, it is nearly impossible to measure the pyridine nucleotide levels under real‐time conditions in different cells (e.g., mesophyll, epidermis, and leaf guard cells) or subcellular compartments (e.g., plastids and cytosol). This has been a hurdle to plant researchers attempting to draw concrete conclusions when studying the redox status in plant cells. Until recently, the development of genetically encoded ratiometric fluorescent protein biosensors has ushered a new era for *in planta* detection of pyridine nucleotides at the subcellular levels. Despite the fact that Peredox‐mCherry, a NADH/NAD^+^ biosensor, was first reported in *Arabidopsis thaliana* (thereafter, Arabidopsis), no further characterization information was available at that time (Wagner et al., 2019). SoNar and iNAPs were the first pyridine nucleotide biosensor variants reported in a plant context with a detailed sensor characterization output (Lim et al., 2020), and later the characterization of Peredox‐mCherry in Arabidopsis was reported (Steinbeck et al., 2020). Recently, a newly developed NADP(H) biosensor, NERNST, was also reported in the Arabidopsis context (Molinari et al., 2023). So far, we introduced the pyridine nucleotides ratiometric fluorescent protein biosensors, namely, SoNar (Zhao et al., 2015) and iNAPs (which consist of different affinity biosensors, iNAP1 and iNAP4) (Tao et al., 2017) in the Arabidopsis plant to measure the dynamic changes of NADPH levels (iNAP1 and iNAP4) and NADH/NAD^+^ ratios (SoNar) in the chloroplasts and cytosol of mesophyll cells; and in the chloroplasts of guard cells (Lim et al., 2020, 2022). Using the SoNar and iNAPs biosensors, we resolved some scientific questions that have been unclear for decades. We revealed that in Arabidopsis, photorespiration provides a large amount of NADH to mitochondria during illumination. Subsequently, the malate‐OAA shuttle transports excess NADH from mitochondria to the cytoplasm (Lim et al., 2020). Apart from that, we also showed that guard cell chloroplasts and mesophyll cell chloroplasts acquire ATP and carbohydrates differently due to the limited photosynthetic capacity of guard cell chloroplasts (Lim et al., 2022). This demonstrates that the current genetically encoded ratiometric fluorescent protein biosensor technology can overcome existing bottlenecks in real‐time metabolite detection in plants.

SoNar and iNAPs biosensors were engineered from *Thermus aquaticus* Rex (T‐Rex) repressor, which is linked with a circularly permuted yellow fluorescent protein and are capable of sensing the redox states of pyridine dinucleotides. These biosensors possess many desirable properties, such as strong fluorescence, fast response, high specificity, and large dynamic range (Tao et al., 2017; Zhao et al., 2015). Similar to SoNar, Peredox‐mCherry was designed from the T‐Rex NAD(H) binding domain which is linked with the blue‐green fluorescent protein tSapphire and a mCherry fused at the C terminus (Hung et al., 2011). Despite being a pH‐insensitive biosensor, the slow dissociation rate of NADH from Peredox‐mCherry makes it difficult to interpret rapid redox changes during *in planta* experiments (Steinbeck et al., 2020), while for SoNar and iNAPs, their pH sensitivity at one of the excitation wavelengths also complicates and limits their applications, as an independent transgenic line expressing a control biosensor (iNAPc) is needed for post‐experimental pH normalization (Lim et al., 2022; Tao et al., 2017; Zhao et al., 2015). The requirement of post‐experimental pH normalization hinders the application of these biosensors, as visualization and comparison of dynamic changes of the pyridine nucleotide redox status in adjacent cells or individual organelles within the same cells are not possible.

To overcome the existing shortcomings of these first‐generation biosensors, we fused mCherry to the N terminus of the biosensors, as previously reported in mammalian cells, allowing ratiometric and pH‐resistant measurements (Tao et al., 2017). We further validated their applications *in vitro* and in Arabidopsis pollen tubes (Liu et al., 2022). Using these improved biosensor variants, we employed them to Arabidopsis whole plants under the control of Cauliflower mosaic virus (CaMV) 35S promoter and reported it in this study. The introduction of pH‐insensitive fluorophore (mCherry) that is reported to be stable from pH 6 to pH 9, and its fusion to the N terminus of the first‐generation SoNar and iNAPs biosensor origin thereby eases the pH limitation (Doherty et al., 2010; Shaner et al., 2004; Tao et al., 2017). These second‐generation biosensors allow us to instantly visualize the changes in NADH/NAD^+^ ratio and NADPH concentration at subcellular levels in compartmentalized plant cells without any need for post‐experimental pH normalization (Liu et al., 2022). Given this advantage, these second‐generation SoNar (mCherry‐SoNar) and iNAPs (mCherry‐iNAPs) biosensors outperform current methods including *in vitro* methods which require extraction of metabolites from tissue prior to taking measurements, and *in vivo* methods which are either slow to respond to NADH (Peredox‐mCherry) or are pH‐sensitive (SoNar and iNAPs).

Here, we further introduced and reported these biosensors into plastids and cytosol of the whole plants of Arabidopsis under the control of the CaMV 35S promoter and illustrated how to monitor real‐time in planta dynamic changes of NADPH and NADH/NAD^+^ ratio in these transgenic lines using confocal microscope, spinning disk microscope, and fluorometer plate reader. We also deposited the first and second generation of transgenic biosensor lines at the Arabidopsis Biological Research Centre (ABRC) (https://abrc.osu.edu/) for further use by plant researchers (Table S2). We anticipate that this second‐generation NADPH (mCherry‐iNAPs) biosensor and NADH/NAD^+^ (mCherry‐SoNar) redox biosensor will be very useful for plant researchers for real‐time monitoring of pyridine nucleotides in more follow‐up studies.

## RESULTS

### Transgenic Arabidopsis lines expressing mCherry‐SoNar/iNAPs biosensors under the control of CaMV 35S promoter

**Figure 1:**
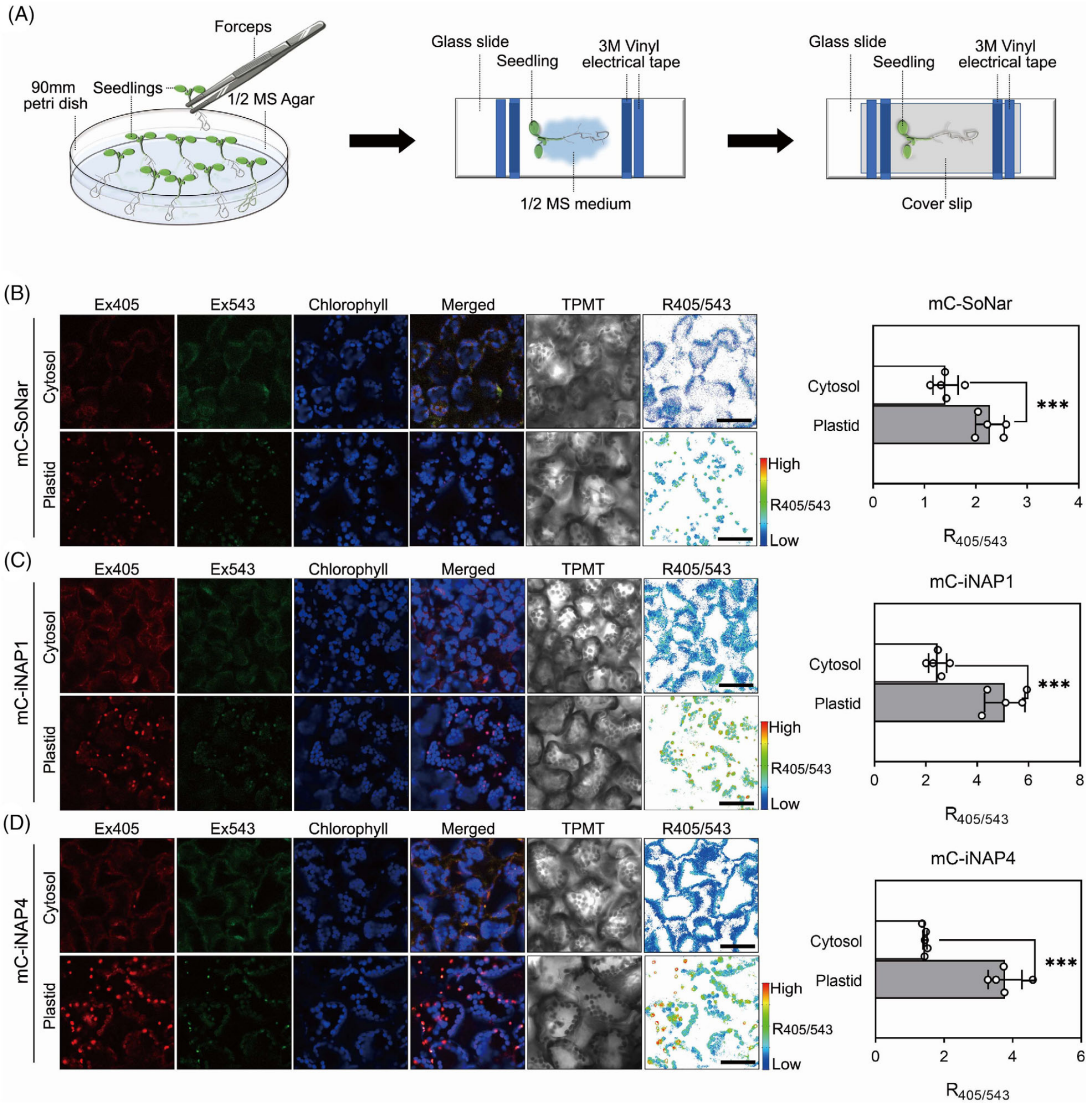
mC‐SoNar and mC‐iNAPs expression in Arabidopsis mesophyll cells under the control of CaMV 35S promoter. (A) Schematic of seedling sample setup: Select the 10‐day‐old seedlings and place them on an imaging chamber with ½ MS medium. Seal the imaging chamber with a coverslip and 3 M vinyl electrical tape before imaging. Subcellular localization of (B) mC‐SoNar, (C) mC‐iNAP1, and (D) mC‐iNAP4 expressed in mesophyll cell cytosol or plastid was verified by fluorescence imaging using confocal microscope (dual excitation at 405 and 543 nm, and emission at 520 ± 16 and 609 ± 25 nm for biosensors, and excitation at 488 nm, emission at 610 nm for chlorophyll). Black scale bar, 50 μm. Comparison of the ratios of mC‐SoNar or mC‐iNAPs between the cytosol and chloroplasts in mesophyll cells (unpaired t‐tests, two‐tailed at ***P < 0.001; *n* = 5; mean ± SD). All the ratio images are presented in pseudocolor, where red corresponds to high NADH/NAD^+^ ratios and high NADPH levels. TPMT, transmitted light detector; mC, mCherry.

Due to the different concentrations of NAPDH in each plant subcellular compartment (Lim et al., 2020), we employed different NADPH affinity biosensors, named mCherry‐iNAP1 (higher affinity), mCherry‐iNAP4 (lower affinity), and NADH/NAD^+^ biosensor mCherry‐SoNar into Arabidopsis ecotype Columbia 0 (Col‐0) background. To expand the use of these improved biosensor variants in plants, here, we exploited these biosensors into the plastids and cytosol of the other plant tissues under the control of the CaMV 35S promoter. As previously reported, we failed to introduce the first‐generation biosensor into mitochondria of WT and suppressor of gene silencing 3–13 (*sgs3‐13*) using the mitochondrial targeting presequence (Lim et al., 2020). Hence, in this study, we did not generate any mitochondrial tagged biosensor plants. To observe the fluorescence intensity of the biosensor transgenic lines under confocal microscopy, we first mounted 10‐day‐old seedlings on glass slides and confirmed their colocalization (Figure 1A–D). Fluorescent signals in the peripheral cytoplasmic region clearly indicated the expression of biosensors in the cytoplasm (Figure 1B–D). We also targeted the mCherry‐SoNar and mCherry‐iNAPs biosensors into plastid stroma using the chloroplast transketolase transit peptide (TKTP) of *Nicotiana tabacum* (Figure 1B–D) and confirmed the colocalization of the biosensor signals with the chlorophyll fluorescence in cotyledons (Figure 1B–D).

As expected, observations of the second‐generation biosensor seedling were corroborated with those of the first‐generation biosensors, where both NADH/NAD^+^ ratio and NADPH levels in the stroma of mesophyll cells were higher than those in the cytosol (Figure 1B–D). Like mesophyll, the pollen tube revealed a higher NADH/NAD^+^ ratio and NADPH level in the plastid stroma than in the cytosol (Figure S1A,B). The colocalization of the plastid in pollen tube was also confirmed with the pollen plastid marker, a filamenting temperature‐sensitive mutant Z (FtsZ1) fused with mCerulean fluorescent protein in pollen tubes (Figure S1C–E). Interestingly, biosensor signals were also observed in the chloroplasts of pavement cells because they were located very close to the mesophyll layer (Figure S2A–C). To date, the mechanisms regulating chloroplasts and their redox status in pavement cells remain poorly understood. It is believed that the plastid types identified in the tissue are associated with specific redox properties, and our biosensor plants may provide a better understanding of NADH/NAD^+^ and NADPH pools in the pavement cell chloroplasts studies.

To evaluate the validity of the collected emission spectra for plants expressing mCherry‐SoNar and mCherry‐iNAPs, we scanned the emission range for mCherry‐SoNar and mCherry‐iNAPs from 424 to 657 nm and 550 to 686 nm in 21‐day‐old seedlings under the excitation wavelengths of 405 nm and 543 nm, respectively (Figure S3A–D). Note that excitation at 543 nm alone results in auto chlorophyll fluorescence starting at an emission wavelength of 638 nm, thus it is necessary to make sure that the emission filter wavelength is set to be less than 638 nm during the seedling imaging process (Figures S3B,D). On the other hand, we also collected the data using wild‐type plants as a control, and the experiment was performed in parallel with all cytosolic/TKTP‐mCherry‐SoNar and mCherry‐iNAPs plants (Figure S3A–D). Notably, all the plants expressing mCherry‐SoNar and mCherry‐iNAPs showed significantly higher fluorescence intensities compared to the wild‐type plants, indicating that the biosensors have been successfully expressed in Arabidopsis.

**Figure 2:**
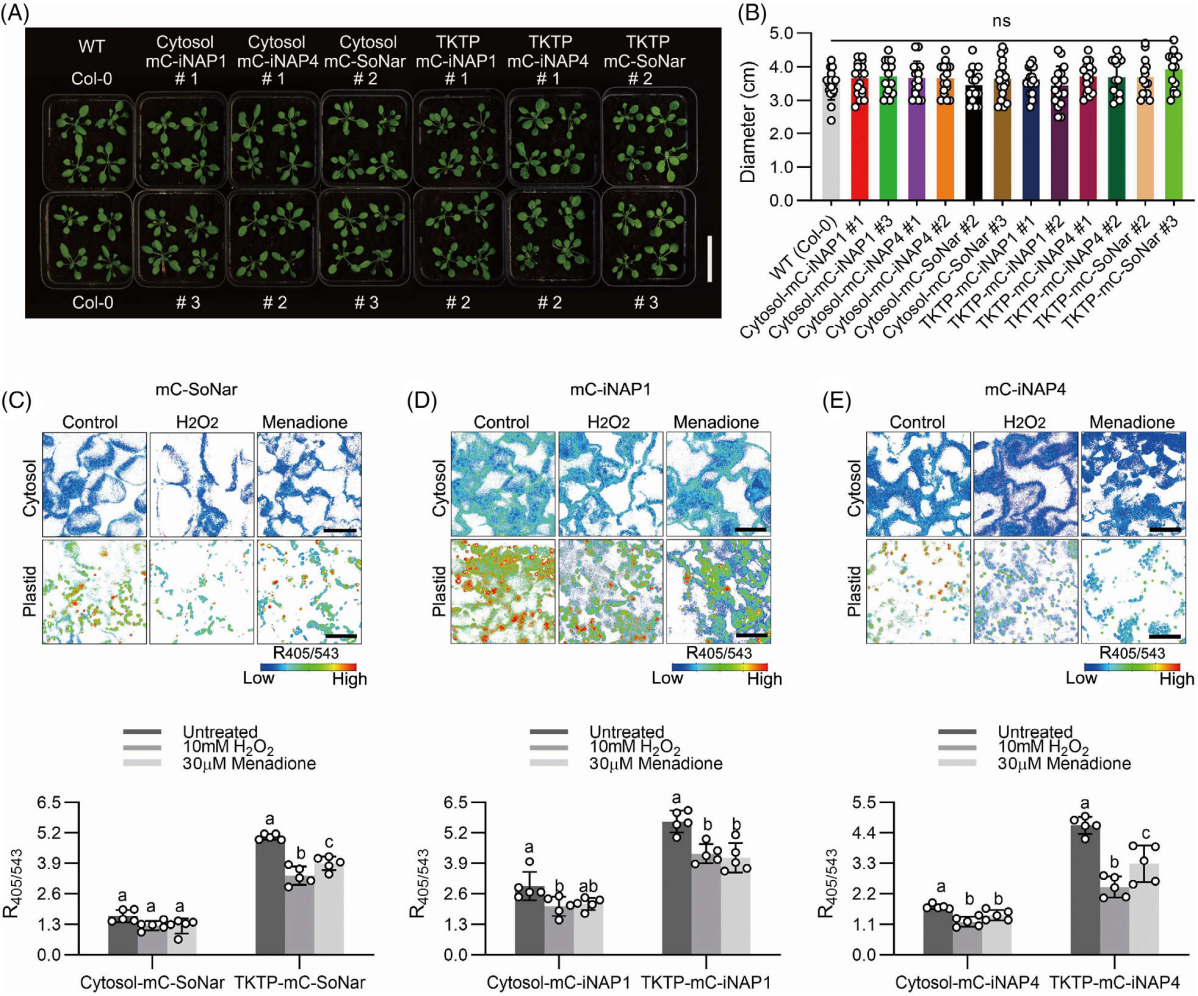
Overview of the plant sizes of 3‐week‐old plants expressing various biosensors under the control of CaMV 35S promoter. Effects of oxidizing agents on cytosol and plastid stroma (TKTP) NADH/NAD^+^ ratios and NADPH levels in 10‐day‐old seedlings. (A) Phenotype of 3‐week‐old plants of different independent lines (Cytosol‐mC‐iNAP1 #1 and #3; Cytosol‐mC‐iNAP4 #1 and #2; Cytosol‐mC‐SoNar #2 and #3; TKTP‐mC‐iNAP1 #1 and #2; TKTP‐mC‐iNAP4 #1 and #2; TKTP‐mC‐SoNar #2 and #3) were compared with that of the wild‐type (Col‐0) plants. White scale bar, 4.25 cm. (B) Plant diameters of the 3‐week‐old plants were measured, there are no significant differences among the lines as determined by Tukey's HSD (*P* < 0.05); *n* > 14 plants; mean ± SD; mC, mCherry; ns, non‐significant. Effects of 10 mm H_2_O_2_ and 30 μm menadione on cytosol and plastid of (C) mC‐SoNar, (D) mC‐iNAP1, and (E) mC‐iNAP4 biosensor ratios in cotyledon mesophyll cells of 10‐day‐old seedlings. Ratio images are presented in pseudocolor where high R405/543 ratios (red) correspond to high NADH/NAD^+^ ratios or NADPH levels. Treatments with significant differences as determined by Tukey's HSD (*P* < 0.05) are indicated with different letters; *n* = 5; mean ± SD; scale bars, 50 μm.

To examine if the expression of biosensors affects plant growth, we monitored the growth phenotypes of two independent biosensor lines of each biosensor in each compartment (Figure 2A,B). All transgenic lines displayed similar phenotypes to wild‐type plants. The rosette diameter did not show significant differences among the lines, indicating that stable expression of these biosensors in Arabidopsis did not lead to any detrimental growth phenotypes (Figure 2B). To further validate the *in planta* responses of mCherry‐SoNar and mCherry‐iNAPs biosensor lines, we treated 10‐day‐old Arabidopsis seedlings with the oxidizing agents (Figure 2C–E). The application of exogenous menadione and H_2_O_2_ significantly decreased the ratio of NADH/NAD^+^ in plastid stroma, but not in the cytosol (Figure 2C). This could be because the cytosol is more oxidized than the chloroplast stroma, and the basal cytosolic NADH/NAD^+^ ratio was low. By contrast, both mCherry‐iNAP1 and mCherry‐iNAP4 in cytosol and plastid stroma showed a statistically significant reduction in NADPH levels when treated with menadione and H_2_O_2_ (Figure 2D,E). These results are consistent with our previous reports on first‐generation biosensors, particularly the observation of *in planta* NADPH level and NADH/NAD^+^ ratio under the treatment of H_2_O_2_ and menadione (Lim et al., 2020). Taken together, this showed a clear validation of mCherry‐SoNar and mCherry‐iNAPs sensitivity in Arabidopsis.

**Figure 3:**
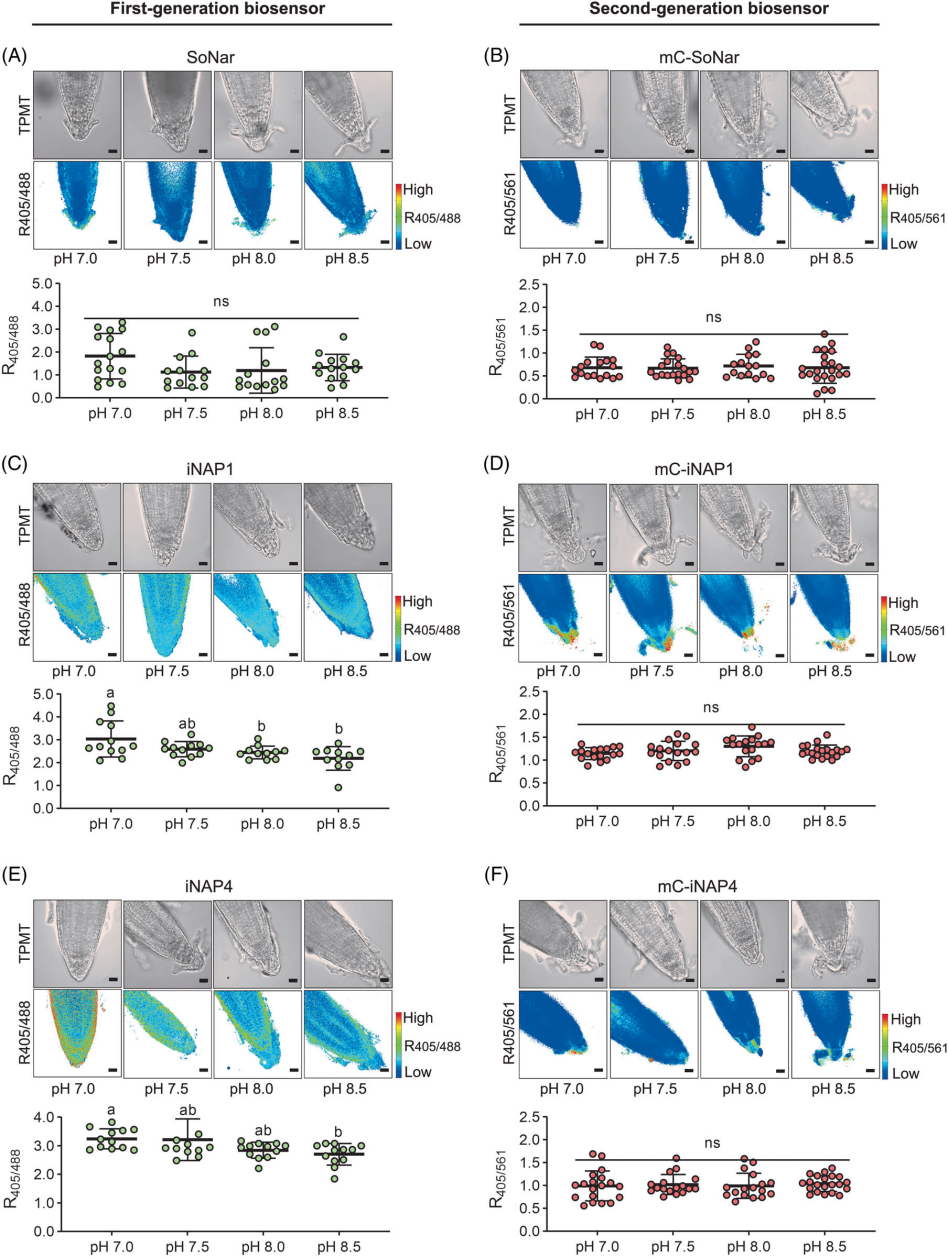
Comparing *in planta* pH sensitivity of the first‐ and second‐generation biosensors. The effects of pH treatment on the root tip ratios of 5‐ to 6‐day‐old seedlings expressing cytosolic biosensors were shown as (A) SoNar, (B) mC‐SoNar, (C) iNAP1, (D) mC‐iNAP1, (E) mC‐iNAP1, and (F) mC‐iNAP4 in half‐strength Murashige and Skoog media adjusted to pH 7.0, pH 7.5, pH 8.0, and pH 8.5, respectively. Ratio images are presented in pseudocolor where high R405/488 or R405/561 ratios (red) correspond to high NADH/NAD^+^ ratios or NADPH levels. Treatments with significant differences as analyzed by Tukey's HSD (*P* < 0.05) are indicated with different letters. *n* > 10 per independent sensor line; mean ± SD; scale bars, 20 μm; mC, mCherry.

To compare the *in planta* pH sensitivity of the first‐ and second‐generation biosensors, we first treated 5‐ to 6‐day‐old seedlings expressing the cytosolic biosensors with different pH of half‐strength Murashige and Skoog (1/2 MS) buffers and immersed them for at least an hour before imaging. Root tips expressing cytosolic mCherry‐SoNar and mCherry‐iNAPs biosensors responded consistently across the pH range 7.0–8.5, showing that these second‐generation biosensors have a better tolerance to pH changes compared to the first‐generation biosensors (Figure 3A–F) and do not require post‐pH normalization.

### Spatiotemporal subcellular changes of pyridine nucleotides in whole plants and various tissues using confocal microscopy

**Figure 4:**
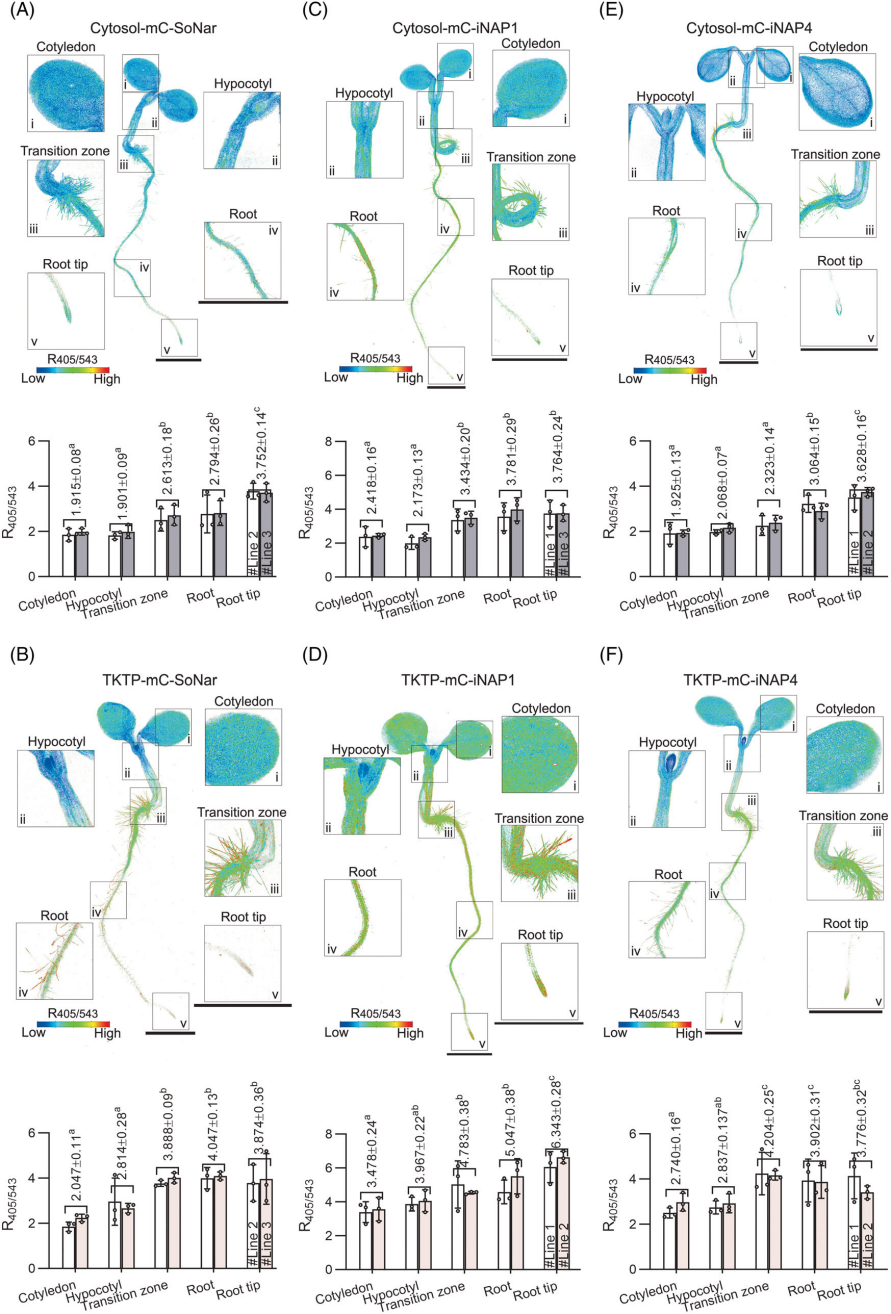
Expression of mC‐SoNar, mC‐iNAP1, and mC‐iNAP4 biosensors in the cytosol and plastid stroma (TKTP) of 5‐day‐old Arabidopsis seedlings. R405/543 ratios obtained from 5‐day‐old seedlings of two independent biosensor lines of (A) cytosolic mC‐SoNar, (B) plastid mC‐SoNar, (C) cytosolic mC‐iNAP1, (D) plastid mC‐iNAP1, (E) cytosolic mC‐iNAP4, and (F) plastid mC‐iNAP4. Fluorescence from dual excitation wavelengths (sequential excitation at 405 nm, emission at 520 ± 16 nm, and excitation at 543 nm, emission at 609 ± 25 nm) was recorded with a confocal microscope laser power at 3%. Enlarged images are i, cotyledon, ii, hypocotyl, iii, transition zone between root and hypocotyl, iv, root, and v, root tip. Ratio images are presented in pseudocolor where high R405/543 ratios (red) correspond to high NADPH levels or NADH/NAD^+^ ratios. Low R405/543 ratios (blue) correspond to low NADPH levels or NADH/NAD^+^ ratios. Tissues with significant differences as determined by Tukey's HSD (*P* < 0.05) are indicated with different letters; *n* = 6 from two independent lines; mean ± SD; scale bars, 2000 μm; mC, mCherry.

To select the suitable NADPH biosensors to be used in different compartments/tissues, we introduced two NADPH biosensors (mCherry‐iNAP1 and mCherry‐iNAP4) with different detection ranges and affinities to NADPH into the cytoplasm and plastid stroma of Arabidopsis. Since mCherry‐SoNar is a biosensor that detects the ratio of NADH to NAD^+^, there is only one version. The levels of pyridine nucleotides in various tissues (e.g., cotyledon, hypocotyl, transition zone between root and hypocotyl, root, and root tips) in the whole Arabidopsis seedlings were examined by tile‐scan under a confocal microscope (Figure 4A–F). The ratios obtained from 5‐day‐old seedlings of two independent lines for each biosensor were nearly consistent, with no statistically significant differences (Figure 4A–F; Figure S4A–F). Tissue‐specific differences in NADH/NAD^+^ ratios and NADPH levels under normal physiological conditions were shown without the need for pH normalization (Figure 4A–F). In particular, all seedlings expressing mCherry‐SoNar and mCherry‐iNAPs biosensors exhibited higher NADH/NAD^+^ ratios and NADPH levels in root (from the differentiated region showing root hairs to the tip including meristem) than the levels in cotyledon and hypocotyl (Figure 4A–F). Additionally, all tissues exhibited lower NADPH levels and NADH/NAD^+^ ratios in the cytosol than in the plastid stroma (Figure 4A–F). Although not shown in the figure, throughout the confocal‐equipped experiments, the autofluorescence channel at the UV spectrum (405 nm excitation and 450 ± 17 nm emission) and the chlorophyll fluorescence (488 nm excitation and 670 ± 30 nm emission) were also set in all seedling experiments to exclude any potential interference from plant phenolics, lignin, and chlorophyll autofluorescence which we did not present here. All these results support the notion that these second‐generation biosensors are suitable for use in Arabidopsis.

### Real‐time dynamic changes of pyridine nucleotides in pollen plastids using spinning disk microscopy

**Figure 5:**
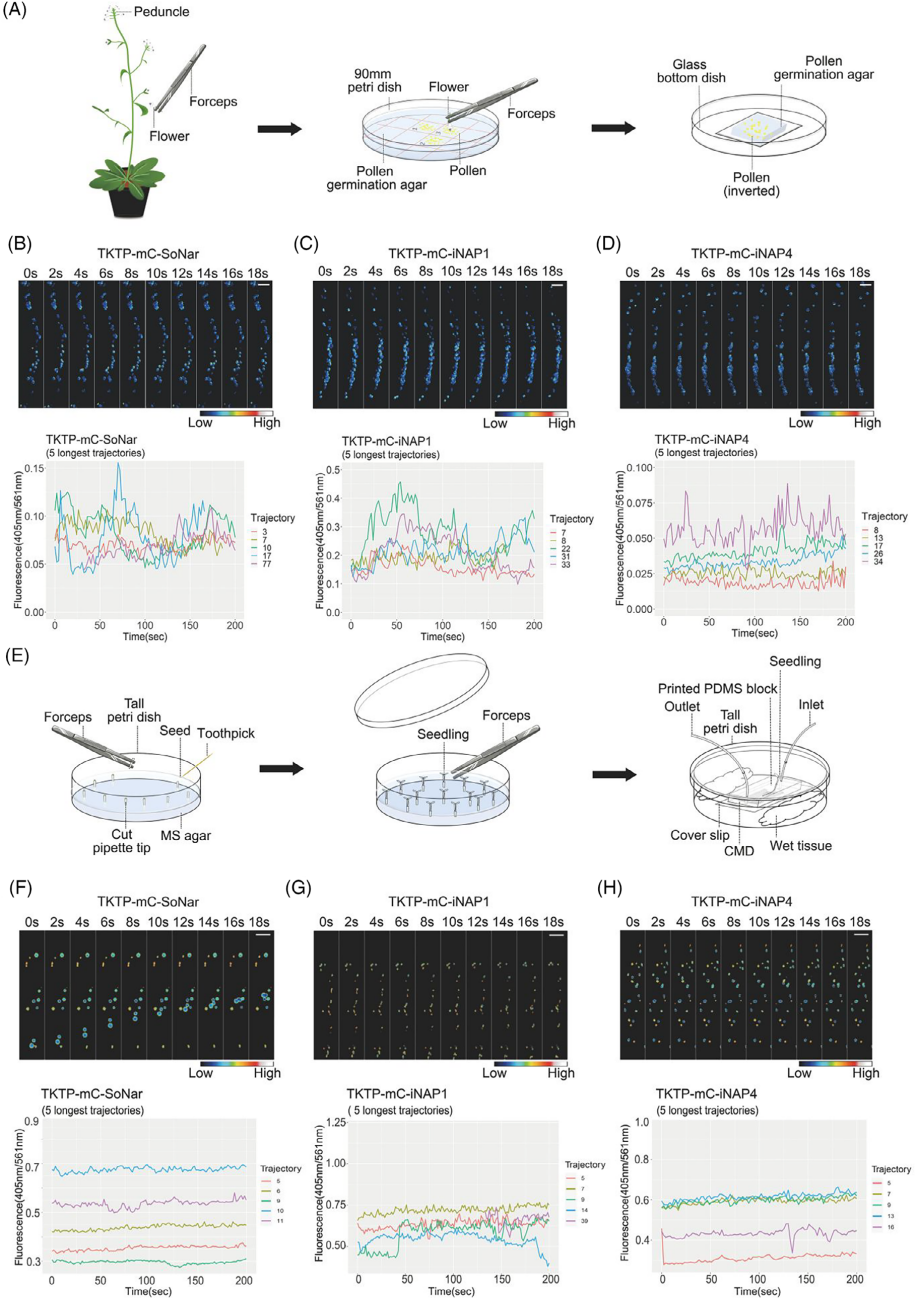
Arabidopsis growing pollen tubes and root hairs expressing mC‐SoNar, mC‐iNAP1, and mC‐iNAP4 biosensors in plastids. (A) Schematic diagram of pollen germination: Use tweezers to pick flowers from the pedicel area, place the flowers upside down to touch the stigma on the pollen germination agar, and incubate at 28°C for 4 h. Place the germinated pollen tubes on the glass bottom cell culture dish and then image. Time‐lapse imaging of pollen plastids with (B) mC‐SoNar, (C) mC‐iNAP1, and (D) mC‐iNAP4 biosensors were taken by the spinning disk microscope, and the first 18 sec of images are presented in this figure. (E) Schematic diagram of root hair sample setup: Germinate seeds on ½ MS medium‐filled tips placed in a tall ½ MS medium‐filled Petri dish. After 4–5 days, verify the growth of the root and transfer seedlings with root meristem close to the tip edge to the coverslip based microfluidic device (CMD). Connect the CMD to a ½ MS liquid medium‐filled syringe placed on a pump, insert the tip containing the seedling, and place the system inside a sterile tall Petri dish with wet tissue to keep the system moist. Let the seedling grow into the system for 4–5 days in a growth chamber, then break the Petri dish apart, place the coverslip on a spinning disk platform, and proceed with imaging. Root hairs expressing (F) mC‐SoNar, (G) mC‐iNAP1, and (H) mC‐iNAP4 in plastids were grown in a microfluidic device in a growth chamber at 21°C 16‐h light/18°C 8‐h dark regime for 5 days. The emission at 520 ± 25 nm excited by 405 nm and at 610 ± 20 nm excited by 561 nm were recorded. The pseudocolor represents a ratio of 405 nm channel to 561 nm channel. A higher ratio (white‐red) represents a higher NADPH level or NADH/NAD^+^ ratio. In the right panels, the five longest trajectories of moving plastids with corresponding biosensors were plotted. Pollen tube scale bars, 20 μm; root hair scale bars, 10 μm; mC, mCherry.

To visualize redox changes in moving organelles, growing pollen tubes were adopted in this study. The preparation of Arabidopsis pollen tubes on the glass bottom dish is shown in Figure 5A. After culturing the mCherry‐SoNar/iNAP1/iNAP4 Arabidopsis pollen tubes for 4 h, a 5‐min time‐lapse of plastid movement and biosensor signals were recorded using spinning disk confocal microscopy by taking advantage of its high scanning speed and low phototoxicity (Video [Link], [Link]). In elongating pollen tubes, most pollen plastids moved out of the grains and distributed in the shank of pollen tubes, traveling directionally forward and backward with dynamic changes in their redox status indicated by the ratios of the biosensor signals (Figure 5B–D). To reveal the relationship between plastid motion and their NADH/NAD^+^ ratio and NADPH level, the movement of a single plastid was tracked with the Trackmate plugin of ImageJ. In the five longest trajectories of moving pollen plastids from the same pollen tube, the signal ratios of TKTP‐mCherry‐SoNar/iNAP1/iNAP4 of individual plastids fluctuated along with their directional movement, confirming the capability of the second‐generation biosensors in tracking rapid real‐time redox changes in individual organelles in a single pollen tube.

### Real‐time dynamic changes of pyridine nucleotides in root hair plastids

As root hairs constitute another model of single‐cell polar tip growth, we recorded the 3D analyses of redox changes in moving plastids in growing root hairs. After growing the transgenic seedlings (mCherry‐SoNar/iNAP1/iNAP4 under the control of CaMV 35S promoter) in a microfluidic device (Figure 5E) (Singh et al., 2021), plastid movements and changes in fluorescence signals of the biosensors were simultaneously tracked for 10 min (imaging every 2 sec) using a spinning disk confocal microscopy (Figure 5F–H, Videos [Link], [Link]). In order to automatically evaluate the plastid movement as well as the ratio of NADH/NAD^+^ and NADPH level, the TrackMate plugin (ImageJ, v. 1.53q) was used. We have presented the first 18 sec of plastid movement visualization and fluctuations of fluorescence ratios between 405/561 nm excitation for the three biosensors (Figure 5F–H; Videos [Link], [Link]). In accordance with previous data (Ovečka et al., 2010), we found a great diversity in plastid sizes as well as in the intensity of biosensor fluorescence signals in a similar way as in pollen tube data (Figure 5B–D).

We have investigated the first 200 sec of the five longest trajectories of moving plastids from the same root hair (Figure 5F–H) and observed a great diversity in the intensity of the fluorescence signals for mCherry‐SoNar compared to mCherry‐iNAP1 and mCherry‐iNAP4. Interestingly, during root hair growth, we also observed important variations in plastid movements showing either slow, ranging from 0.001 to 0.19 μm/s, or fast forward/backward movements ranging from 3.1 to 8.1 μm/s. Thus, during root hair growth, plastids showed rapid dynamic changes in their redox potential as observed in the pollen tubes.

### Simultaneous real‐time measurement of plant tissues using a multi‐well fluorimetry plate reader

**Figure 6:**
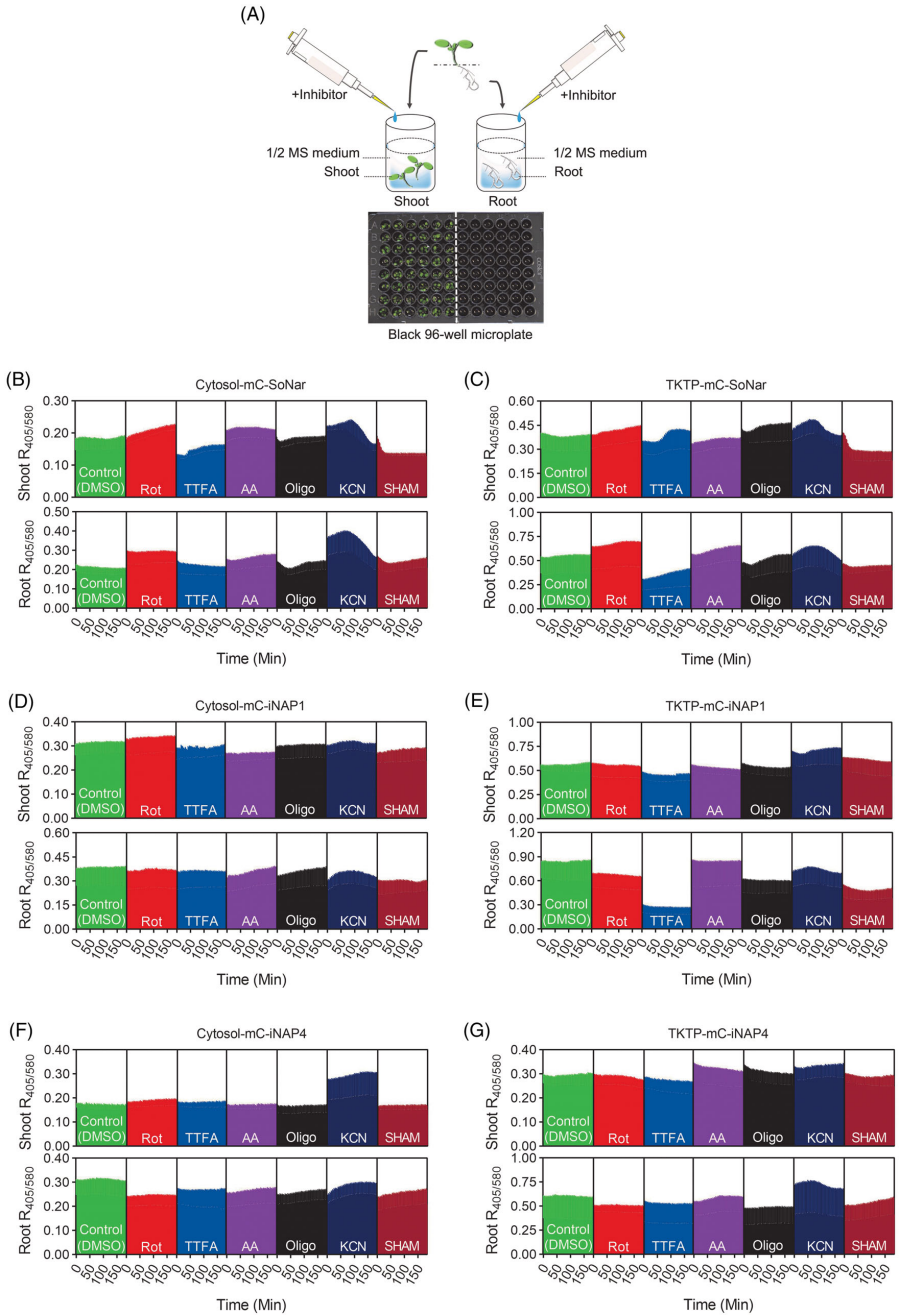
Dynamic responses of cytosolic and stromal NADH/NAD^+^ ratios (mC‐SoNar) and NADPH levels (mC‐iNAP1 and mC‐iNAP4) in seedlings to various inhibitors in black 96‐well microplate. (A) Schematic view of the black 96‐well plate setup: two identical biosensor‐expressing seedlings were detached into shoots and roots, which were then placed into separate wells of a black 96‐well microplate containing ½ MS medium with different inhibitors, including 50 μm rotenone (Rot), 100 μm thenoyltrifluoroacetone (TTFA), 10 μm antimycin A (AA), 10 μm oligomycin (Oligo), 500 μm potassium cyanide (KCN), and 2 mm salicylhydroxamic acid (SHAM). Kinetic responses of biosensor ratios of (B) cytosolic mC‐SoNar, (C) plastid mC‐SoNar, (D) cytosolic mC‐iNAP1, (E) plastid mC‐iNAP1, (F) cytosolic mC‐iNAP4, and (G) plastid mC‐iNAP4 expressing seedlings to different inhibitors were recorded up to 3 h in the dark. *n* = 6 (with 12 seedlings); mean ± SD; mC, mCherry. In panel (b–g), the upper and lower panels showed the shoot and root data, respectively.

Confocal microscope and spinning disk microscope do not allow simultaneous measurement of a large number of plant samples. Here, we employed a multi‐well fluorimetry plate reader to study the kinetic changes of NADH/NAD^+^ ratios and NADPH levels in the plastids and cytosol of the shoots and roots of Arabidopsis in response to mitochondrial electron transport chain (mETC) inhibitors. The kinetic changes of biosensor ratios in 10‐day‐old seedlings under the influences of rotenone, TTFA, antimycin A, oligomycin, KCN, and SHAM, were monitored for 3 h using a microplate fluorometer reader (Figure 6A–G).

As expected, the external addition of rotenone and TTFA resulted in a gradual increase of NADH/NAD^+^ ratio in Arabidopsis shoot cytosol and plastids, respectively, throughout the 3 h of observation. However, no significant increase in NADPH level was observed, indicating that mitochondrial activities only affect the plastid and cytosolic NADH pools but have less impact on the NADPH pools (Figure 6B–G). Compared to rotenone and the control, the application of TTFA instantly lowered the initial NADH/NAD^+^ ratio in plastid and the cytosol of the shoots and roots, possibly because of its ability to inhibit succinate dehydrogenase, a key step of the tricarboxylic acid cycle (TCA) in the dark (Figure 6B,C). Oligomycin and SHAM treatments in both cytosolic and plastid of the root and shoot resulted in biphasic changes in the NADH/NAD^+^ ratio which were initially quickly decreasing, then either slowly increasing again or remaining stationary (Figure 6B,C). A small increment of NADH/NAD^+^ ratio in plastid was detected in antimycin A treated shoot and root (Figure 6C). The KCN treatment affects both the NADH/NAD^+^ and NADPH levels. Interestingly, all the KCN‐treated seedlings showed a biphasic pattern with both NADPH and NADH/NAD^+^ ratios slowly increasing at first, followed by a quick decrease as cyanide effectively inhibited cytochrome c oxidase.

On the other hand, the shoots of both TKTP‐mCherry‐iNAP1 and TKTP‐mCherry‐iNAP4 biosensor lines demonstrated a decrease in NADPH level when treated with mETC inhibitors (except KCN), but this trend was not seen in cytosolic biosensors (Figure 6D–G). Collectively, the above observations are in agreement with the results of our first‐generation biosensors, showing that the inhibition of mETC under dark conditions led to the accumulation of NADH in the cytoplasm and plastids. These data indicate that the NADH/NAD^+^ ratios in plastids, cytosol, and mitochondria are interconnected. The observation that the increases in cytosolic and plastid NADH/NAD^+^ ratios upon treatments of mETC inhibitors were not accompanied by simultaneous changes in the NADPH levels in these two compartments, indicates that NADH/NAD^+^ ratios and NADPH concentrations in these compartments are differentially regulated (Figure 6B–G).

## DISCUSSION

Existing pyridine nucleotide assays have been widely used but are unable to fully reveal the subcellular distribution of NADPH and NADH/NAD^+^ in plant cells. Hence, plant scientists always seek a better way to resolve this limitation. Although the SoNar and iNAPs genetically encoded fluorescent biosensors were originally engineered for mammalian cells (Tao et al., 2017; Zhao et al., 2015). Due to their high stability, responsiveness, and suitable detection ranges for physiological concentrations of plant cells, we applied the first‐generation SoNar and iNAPs biosensors for plant research (Haber et al., 2021; Lim et al., 2020, 2022). In fact, the major drawback of the first‐generation SoNar and iNAPs biosensors is their pH sensitivity during excitation at 488 nm, which limits the application of these biosensors in a pH‐fluctuating environment (Lim et al., 2020). To overcome the pH limitation, a mCherry fluorophore was fused into the N terminus of first‐generation SoNar and iNAPs as previously reported (Liu et al., 2022; Tao et al., 2017). It was reported that the recombinant protein of mCherry‐SoNar/iNAPs behaves stably between pH 7.0 and 8.5 (Liu et al., 2022). Thus, in this study, we introduced the second‐generation mCherry‐SoNar and mCherry‐iNAPs biosensors into Arabidopsis under the control of CaMV 35S promoter and validated their applications. Since the pH of the cytoplasm is generally around 7.3, and the pH of the plastid matrix is around 7.2 (Shen et al., 2013), the pH stability of mCherry from pH 6 to 9 allows us to monitor the dynamic changes of NADPH levels and NADH/NAD^+^ ratios in individual cells (Figure 4A–F) and individual organelles in the same cells without the need for pH normalization (Figure 5B–D, F–H). The advantage of these biosensors is that they are easy to use with most fluorescence equipment by matching their fluorophores. The fluorescence intensities for the second generation of mCherry‐SoNar and mCherry‐iNAPs can be collected using blue filter sets and mCherry filter sets. More importantly, we can easily monitor the redox changes in different cells and tissues using various desired fluorescence equipment. As shown in this study, a confocal microscope (Figures 1, 2, 3, 4; Figures S1–S4), spinning disk confocal microscope (Figure 5; Videos [Link], [Link]), and fluorometer plate reader (Figure 6) can be used to work with fluorescent protein transgenic lines for a variety of applications. However, it is worth noting that the ratios collected from different instruments cannot be compared, as different instruments may report different fluorescence intensities, resulting in different ratios (Bajar et al., 2016). Although there were small changes observed in dissociation constant (*K*_d_) in the second‐generation biosensors, the mCherry‐iNAP1 (from 0.29 to 0.94 μm) and mCherry‐iNAP4 (from 33 to 39 μm), when compared to the first‐generation biosensors (Lim et al., 2020; Liu et al., 2022). The changes in the *K*_d_ of the biosensors may be due to changes in excitation properties (Müller‐Schüssele et al., 2021). Our validation results suggested that the second‐generation biosensors are still suitable for use in Arabidopsis because they remain within the physiological ranges (Figures 1B–D, 2C–E). In view of this, our biosensor seedling validation data may serve as a guide and control when conducting experiments.

So far, many other metabolite biosensors have been developed and characterized for use in Arabidopsis. Concisely, a summary of available biosensors used in Arabidopsis is provided in the Supporting Information Table S1. The advantage of using ratiometric fluorescent protein biosensors is that their emission ratios are independent of the biosensor protein concentrations expressed in different tissues, cells, or compartments. Similar to the other genetically encoded ratiometric biosensors in plant cells (Table S1), ratiometric changes in the fluorescence intensities emitted by the biosensors correlate with the changes in target metabolites in the plant cells. The change in the fluorescence intensity of the biosensor solely depends on the binding of the target molecule (Bajar et al., 2016; Rowe & Jones, 2021). We also successfully studied these pyridine nucleotide biosensors in pollen tubes (Figure 5B–D; Figure S1, Videos [Link], [Link]) and root hair (Figure 6F–H, Videos [Link], [Link]). Reliable data on the redox state concerning pollen tube and root hair growth are largely lacking, but it seems critical if we can understand the mechanisms of the pollen tube and root hair growth and their energy conversion. Of interest as well is to understand the correlation between plastid movement and metabolism as suggested by our results during apical cell growth. Notably, with the help of these mCherry‐SoNar and mCherry‐iNAPs biosensors, we have recently pinpointed the energy sources that support pollen tube growth and fatty acid biosynthesis in pollen plastids (Liu et al., 2022). To further understand the pyridine nucleotide distribution in the whole plants, we further generated transgenic lines that express these biosensors *in planta*. Our findings showed that whole plant cells have relatively lower NADPH levels and NADH/NAD^+^ ratios in the cytosol than in the plastid stroma, which coincides with our observations in mesophyll cells (Figures 1B–D, 4A–F). Interestingly, ATP concentration has been reported to have an inverse distribution, where a high ATP level was detected in the cotyledon, but it was lower in the root (De Col et al., 2017; Voon et al., 2018). This may imply that NADH oxidation in different plant tissues is closely related to ATP production. Consistently, the same observation was also reported in Arabidopsis using the Peredox‐mCherry biosensor (Steinbeck et al., 2020), inferring that the high NADH/NAD^+^ ratios in the root could be interpreted as the lower mETC activity in the root. We sought to further understand the specificity of the NADH/NAD^+^ and NADPH pools using the second‐generation mCherry‐SoNar and mCherry‐iNAPs biosensors, complementing our previous first‐generation biosensors. Our work revealed that different plant tissues have distinct NADH/NAD^+^ ratios and NADPH levels.

In summary, the rapid responses of mCherry‐SoNar and mCherry‐iNAPs and the pH insensitivity of their biosensor ratios make them ideal *in planta* biosensors of pyridine nucleotides to date. To make the seeds available for plant researchers, we deposited the first‐generation and second‐generation biosensors of Arabidopsis transgenic lines either expressed in the cytosol or targeted to plastids under the control of CaMV 35S promoter and LAT52 promoter at the ABRC (Table S2) and detailed methods are also included in Supplemental Materials and Methods. The expanded application of the second‐generation SoNar and iNAPs biosensors in different plant organelles and tissues will allow us to discover and further understand the novel redox response of Arabidopsis under biotic and abiotic stresses. Combining different metabolite biosensors with fully processed image data will open up many exciting possibilities for future works in plant science, for instance, correlating the growth and physiology of plant tissues, or investigating the connection of cellular organelles' energy source mechanisms. These sorts of approaches can yield impressive results, allowing the *in planta* metabolic pathway to be recapitulated and studied in greater depth.

## MATERIALS AND METHODS

### Plasmid construction and generation of transgenic plant lines

To construct the second‐generation biosensor vectors, the iNAPs and SoNar cDNAs were amplified from the first‐generation pENTR‐iNAP1, pENTR‐iNAP4, and pENTR‐SoNar vectors (Lim et al., 2020) with a 34‐bp overhang forward primer carrying a BamHI restriction site, whereas the mCherry gene was amplified from a pENTR‐mCherry‐TOC33 vector with a 24‐bp overhang reverse primer carrying a BamHI restriction site. This 20‐aa peptide linker (GGSGG)_4_ was created via the overhang of the forward and reverse primer from SoNar/iNAPs and mCherry, respectively. The mCherry and SoNar/ iNAPs cDNAs were ligated and cloned into the EcoRI/HindIII sites of a pRSETb vector. cDNA encoding mCherry‐SoNar and mCherry‐iNAPs were amplified from the pRSETb‐mCherry‐SoNar and mCherry‐iNAPs vectors by PCR and cloned into a modified Gateway pENTR/D‐TOPO vector (Invitrogen, Carlsbad, CA, USA) using the ClonExpress II One Step Cloning Kit (Vazyme, Nanjing, China). To construct vectors that express the biosensors in pollen tubes, the LAT52 promoter was amplified from the pGTkan3‐pLAT52 vector, a kind gift from Prof. Li‐Qing Chen of the University of Illinois Urbana‐Champaign and cloned into the modified Gateway pENTR/D‐TOPO vector using ClonExpress II One Step Cloning Kit (Vazyme). cDNA of mCherry‐SoNar and mCherry‐iNAPs was amplified and cloned into a modified Gateway pENTR/D‐TOPO vector carrying LAT52 promoter. The sequence of *Nicotiana tabacum* chloroplast transketolase transit peptide (TKTP) was fused to the N terminus of the mCherry‐SoNar and mCherry‐iNAPs. For cytosolic targeting biosensor lines, the unfused versions were used. Primer sequences are listed in the Supporting Information Table S3. All the constructs were confirmed by nucleotide sequencing by BGI, China. All cDNA constructs in pENTR vectors were then transferred to the plant transformation Gateway destination vectors, pEarleyGate302 (LAT52 promoter) and pH7WG2 (CaMV 35S promoter) vectors. The pEarleyGate302 and pH7WG2 carrying biosensor vectors were transformed into Arabidopsis (ecotype Columbia, Col‐0) using the *Agrobacterium tumefaciens* strain GV3101‐mediated floral dip method. Positive transformants were screened using a fluorescence stereomicroscope (Olympus, SZX16). Next, we kept independent line of the first‐generation transformed plants (T1) with good fluorescence signals and used the T1 plants to collect seeds that were considered as second‐generation plants (T2). Most T2 seeds were heterozygous. T2 plants were then regrown and compared the phenotypes with wild‐type plant. T2 plants with normal growth conditions and good fluorescence signals were selected for subsequent experiments.

### Arabidopsis seedling, root hair, and pollen tube cultivation

For 5‐ and 10‐day‐old seedlings, seeds were germinated on plates of full‐strength MS medium (Murashige & Skoog, 1962) supplemented with 2% (w/v) sucrose and 1% (w/v) Phytagel, and stratified in the dark at 4°C for 2 days before being transferred to a long‐day condition culture room of 16 h with 120–150 μmol photon m^−2^ sec^−1^ at 22°C and 8 h in darkness at 18°C. For 3‐ to 4‐week‐old plants, seedlings were transplanted from plates into peat substrate (Jiffy, Zwijndrecht, the Netherlands) and grown in a plant growth chamber (Panasonic, MLR‐352) with the same growth conditions before mounting onto slides for microscopy imaging acquisition (Video S7).

For pollen tube cultivation, fresh flowers from one‐and‐a‐half‐month‐old plants were taken and mature pollen were dusted on the surface of a solid pollen germination medium supplemented with 1 mm Ca (NO)_3_, 1 mm Ca (Cl)_2_, 1 mm MgSO_4_, 0.01% (w/v) Boric acid, 18% (w/v) sucrose, and 0.8% (w/v) agarose with pH 7.5 adjusted by using KOH (Video S8). Germination medium with pollen was incubated at 28°C for 3–4 h before observation under the confocal microscope or Perkin Elmer Ultraview VOX Spinning Disk Confocal Microscope.

For root hair cultivation, seedlings were grown and transferred into the microfluidic devices in ½ MS medium supplemented with 0.5% (w/v) sucrose as described (Singh et al., 2021). Root hair analyses were performed on seedlings after 5 days of growth in microfluidic device using Zeiss Axio Observer Z1 Spinning Disk Confocal Microscope.

### Confocal microscope and ratiometric image analysis

Confocal images were captured with 10×/0.45 Ph1 or 40×/1.4 oil DIC lenses in multitrack mode using a Zeiss LSM710 or LSM 980 confocal microscope (Carl Zeiss Microscopy, Germany). The imaging setup of the biosensor seedlings was as previously described (Lim et al., 2020; Wagner et al., 2015). Plants expressing the second‐generation biosensors, mCherry‐iNAPs and mCherry‐SoNar, were excited sequentially at 405 and 543 or 561 nm, and emission was detected at 520 ± 16 nm and 609 ± 25 nm, respectively (Table S4). The ratio of mCherry‐iNAPs and mCherry‐SoNar represented by R_405/543_ or R_405/561_ was the raw ratio of emission excited at 405 and 543 or 561 nm. Confocal images were processed using a custom MATLAB‐based suite (Fricker, 2016) and the images were analyzed on a pixel‐by‐pixel basis (Table S5, Video S9).

### Spinning disk confocal microscope

Time‐lapse imaging of growing pollen tubes expressing TKTP‐mCherry‐SoNar/iNAP1/iNAP4 was acquired every 2 sec using a Perkin Elmer Ultraview VOX Spinning Disk Confocal Microscope equipped with a Hamamatsu C9100‐23B EMCCD camera. A 5‐min real‐time movie of pollen tube growth was taken. Pollen tubes were observed by a 60 × 1.4 oil objective lens and excited at the double excitation of 405 and 561 nm wavelength laser, while emission was detected at 520 ± 25 and 610 ± 20 nm, respectively. The pollen plastid movement was analyzed by Fiji ImageJ (https://imagej.net/Fiji) software as well as its Trackmate plugin. The movement speed and ratio change analysis were performed with Rstudio (Video S10).

Time‐lapse imaging of growing root hairs expressing TKTP‐mCherry‐SoNar/iNAP1/iNAP4 was performed using a Zeiss Axio Observer Z1 Spinning Disk Confocal Microscope equipped with a Yokogawa CSU‐X1 head. Z‐stack images were acquired every 2 sec (14–17 slices with 1.5‐μm slice difference per stack). Root hairs were observed with a 63× Plan‐Apochromat 1.40 oil objective under a double excitation at 405 and 561 nm wavelength with a low laser power (15%), while emission was detected at 520 ± 25 and 610 ± 20 nm, respectively. Plastid movement was analyzed using the TrackMate plugin (https://github.com/fiji/TrackMate) from Fiji (ImageJ) software (https://imagej.net/software/fiji/).

### Multi‐well plate reader‐based fluorimetry

Fluorescence intensities of mCherry‐SoNar and mCherry‐iNAPs seedlings were collected using a Cytation 1 Cell Imaging Multi‐mode Reader (BioTek, USA). mCherry‐SoNar and mCherry‐iNAPs were excited with dual wavelengths of 400 ± 10 and 580 ± 25 nm, and the emission wavelength was recorded at 520 ± 25 and 620 ± 10 nm using a black 96‐well microplate (Corning Costar, USA). The internal temperature was kept at 25°C.

### Validation of *in planta* biosensors by treatments with inhibitors and oxidants

To validate the *in planta* responses of the biosensors, 10‐day‐old seedlings expressing the sensors were first detached into shoots and roots. The separated shoots or roots from two seedlings were placed in each well of a black 96‐well microplate and infiltrated in 1/2 MS medium without (DMSO as control) or with the following mETC inhibitors for 5 min: 50 μm rotenone, 100 μm TTFA, 10 μm antimycin A, 10 μm oligomycin, 500 μm KCN, or 2 mm SHAM. The fluorescence intensities of the seedlings in the black 96‐well microplate were detected by the Cytation 1 multi‐mode reader.

For oxidant treatments, 10‐day‐old seedlings were placed in a 24‐well plate and infiltrated with 10 mm H_2_O_2_ or 30 μm menadione for 5 min. The infiltrated seedlings in the transparent 24‐well plate were mounted on a glass slide before observation under a confocal microscope.

### Comparing *in planta* pH sensitivity of the first‐ and second‐generation biosensors

Five‐ to six‐day‐old seedlings expressing cytosolic first‐ and second‐generation SoNar and iNAPs were immersed in the 1/2 MS medium adjusted to pH 7.0, pH 7.5, pH 8.0, or pH 8.5, respectively. After vacuum infiltration for 5 min, all seedlings were immersed in the media with various pHs for at least an hour before imaging under a confocal microscope.

### Statistical data analysis

All data are shown as the means with standard error (mean ± SD). GraphPad PRISM 8 graphing software was used to plot the bar and line graph. The collected data were analyzed for statistical significance using analysis of variance (one‐way ANOVA with post hoc Tukey's HSD test) and paired or unpaired *t*‐test, two‐tailed at *P* < 0.001, *P* < 0.01, and *P* < 0.05 by SPSS (Version 26).

## AUTHOR CONTRIBUTIONS

BLL, SLL, and JL conceptualized and designed the study. SLL, JL, LY, and JYZ participated in the plant material preparation. SLL and JL carried out the seedling and pollen tube experiments and analyzed the data. SG and GD carried out the root hair experiments, GD analyzed the root hair data and prepared the figure. SB prepared the microfluidic chips. BLL, CME, SLL, and JL wrote the manuscript. All authors revised and approved the manuscript.

## CONFLICT OF INTEREST

The authors declare that they have no competing interests.

## OPEN RESEARCH BADGES

This article has earned an Open Materials badge for making publicly available the components of the research methodology needed to reproduce the reported procedure and analysis. All materials are available at https://abrc.osu.edu/ Our seeds have been deposited in ABRC. The accession numbers are listed in Table S2.

## Supporting information


**Video S1.** A 5‐min time‐lapse of pollen tube growth captured on pollen tube expressing TKTP‐mCherry‐SoNar using spinning disk microscopy. Red channel indicates excitation wavelength of 405 nm and emission of 520 ± 25 nm. Green channel indicates excitation wavelength of 561 nm and emission detected at 610 ± 20 nm. 405/561 indicates the ratio images and presented in pseudocolor where high R405/543 ratios (white) correspond to high NADH/NAD^+^ ratios. Low R405/543 ratios (black) correspond to low NADH/NAD^+^ ratios. Scale bar, 10 μm.

**Video S2.** A 5‐min time‐lapse of pollen tube growth captured on pollen tube expressing TKTP‐mCherry‐iNAP1 using spinning disk microscopy. Red channel indicates excitation wavelength of 405 nm and emission of 520 ± 25 nm. Green channel indicates excitation wavelength of 561 nm and emission detected at 610 ± 20 nm. 405/561 indicates the ratio images and presented in pseudocolor where high R405/543 ratios (white) correspond to high NADPH level. Low R405/543 ratios (black) correspond to low NAPDH level. Scale bar, 10 μm.

**Video S3.** A 5‐min time‐lapse of pollen tube growth captured on pollen tube expressing TKTP‐mCherry‐iNAP4 using spinning disk microscopy. Red channel indicates excitation wavelength of 405 nm and emission of 520 ± 25 nm. Green channel indicates excitation wavelength of 561 nm and emission detected at 610 ± 20 nm. 405/561 indicates the ratio images and presented in pseudocolor where high R405/543 ratios (white) correspond to high NADPH level. Low R405/543 ratios (black) correspond to low NAPDH level. Scale bar, 10 μm.

**Video S4.** A 10‐min time‐lapse of root hair growth captured on seedling expressing TKTP‐mCherry‐SoNar using spinning disk microscopy. Red channel indicates excitation wavelength of 405 nm and emission of 520 ± 25 nm. Green channel indicates excitation wavelength of 561 nm and emission detected at 610 ± 20 nm. 405/561 indicates the ratio images and presented in pseudocolor where high R405/543 ratios (white) correspond to high NADH/NAD^+^ ratios. Low R405/543 ratios (black) correspond to low NADH/NAD^+^ ratios. Scale bar, 10 μm.

**Video S5.** A 10‐min time‐lapse of root hair growth captured on seedling expressing TKTP‐mCherry‐iNAP1 using spinning disk microscopy. Red channel indicates excitation wavelength of 405 nm and emission of 520 ± 25 nm. Green channel indicates excitation wavelength of 561 nm and emission detected at 610 ± 20 nm. 405/561 indicates the ratio images and presented in pseudocolor where high R405/543 ratios (white) correspond to high NADPH level. Low R405/543 ratios (black) correspond to low NAPDH level. Scale bar, 10 μm.

**Video S6.** A 10‐min time‐lapse of root hair growth captured on seedling expressing TKTP‐mCherry‐iNAP4 using spinning disk microscopy. Red channel indicates excitation wavelength of 405 nm and emission of 520 ± 25 nm. Green channel indicates excitation wavelength of 561 nm and emission detected at 610 ± 20 nm. 405/561 indicates the ratio images and presented in pseudocolor where high R405/543 ratios (white) correspond to high NADPH level. Low R405/543 ratios (black) correspond to low NAPDH level. Scale bar, 10 μm.

**Video S7.** A step‐by‐step guide on how to prepare seedling slide for imaging.

**Video S8.** A step‐by‐step description of pollen collection and pollen tube culture.

**Video S9.** Step‐by‐step instructions on how to use RRA MATLAB software to analyze confocal imaging data.

**Video S10.** A guide to data analysis of pollen tube growth and root hair growth spinning disk imaging data using Fiji and Rstudio.

**Figure S1.** mC‐SoNar and mC‐iNAPs expression in Arabidopsis pollen tubes under the control of LAT52 promoter.
**Figure S2.** Chloroplasts in the pavement cell.
**Figure S3.** Emission spectra of 21‐day‐old plants expressing different biosensors.
**Figure S4.** An overview of the confocal and ratio images of two independent lines for each of the biosensors in 5‐day‐old seedlings.
**Table S1.** Summary of biosensors used in Arabidopsis research.
**Table S2.** Details of genetically encoded pyridine nucleotide biosensor transgenic Arabidopsis lines deposited at ABRC.
**Table S3.** Primer sequences used for plasmid constructions.

**Table S4.** Excitation and emission wavelengths setup for confocal microscopy.
**Table S5.** Custom MATLAB probe parameters.
**Table S6.** Troubleshooting table.

## Data Availability

The authors declare that the main data supporting the findings of this study are available within the article and its Supplementary Information files, methods, and materials. Extra data are available from the corresponding authors upon request.

## References

[tpj16796-bib-0001] Agius, S.C. , Rasmusson, A.G. & Møller, I.M. (2001) NAD(P) turnover in plant mitochondria. Functional Plant Biology, 28, 461–470.

[tpj16796-bib-0002] Bajar, B.T. , Wang, E.S. , Zhang, S. , Lin, M.Z. & Chu, J. (2016) A guide to fluorescent protein FRET pairs. Sensors, 16, 1488. 27649177 10.3390/s16091488 PMC5038762

[tpj16796-bib-0003] Brodelius, P. & Vogel, H.J. (1985) A phosphorus‐31 nuclear magnetic resonance study of phosphate uptake and storage in cultured *Catharanthus roseus* and *Daucus carota* plant cells. Journal of Biological Chemistry, 260, 3556–3560. 3972837

[tpj16796-bib-0004] Considine, M.J. & Foyer, C.H. (2014) Redox regulation of plant development. Antioxidants & Redox Signaling, 21, 1305–1326. 24180689 10.1089/ars.2013.5665 PMC4158970

[tpj16796-bib-0005] De Col, V. , Fuchs, P. , Nietzel, T. , Elsässer, M. , Voon, C.P. , Candeo, A. et al. (2017) ATP sensing in living plant cells reveals tissue gradients and stress dynamics of energy physiology. eLife, 6, 26770. 10.7554/eLife.26770 PMC5515573 28716182

[tpj16796-bib-0006] Doherty, G.P. , Bailey, K. & Lewis, P.J. (2010) Stage‐specific fluorescence intensity of GFP and mCherry during sporulation in Bacillus subtilis. BMC Research Notes, 3, 1–8. 21073756 10.1186/1756-0500-3-303 PMC2994887

[tpj16796-bib-0007] Fernie, A.R. , Carrari, F. & Sweetlove, L.J. (2004) Respiratory metabolism: glycolysis, the TCA cycle and mitochondrial electron transport. Current Opinion in Plant Biology, 7, 254–261. 15134745 10.1016/j.pbi.2004.03.007

[tpj16796-bib-0008] Foyer, C.H. , Noctor, G. & Hodges, M. (2011) Respiration and nitrogen assimilation: targeting mitochondria‐associated metabolism as a means to enhance nitrogen use efficiency. Journal of Experimental Botany, 62, 1467–1482. 21282329 10.1093/jxb/erq453

[tpj16796-bib-0009] Fricker, M.D. (2016) Quantitative redox imaging software. Antioxidants & Redox Signaling, 24, 752–762. 26154420 10.1089/ars.2015.6390

[tpj16796-bib-0010] Haber, Z. , Lampl, N. , Meyer, A.J. , Zelinger, E. , Hipsch, M. & Rosenwasser, S. (2021) Resolving diurnal dynamics of the chloroplastic glutathione redox state in Arabidopsis reveals its photosynthetically‐derived oxidation. Plant Cell, 33, 1828–1844. 33624811 10.1093/plcell/koab068 PMC8254480

[tpj16796-bib-0011] Heineke, D. , Riens, B. , Grosse, H. , Hoferichter, P. , Peter, U. , Flugge, U.I. et al. (1991) Redox transfer across the inner chloroplast envelope membrane. Plant Physiology, 95, 1131–1137. 16668101 10.1104/pp.95.4.1131 PMC1077662

[tpj16796-bib-0012] Hung, Y.P. , Albeck, J.G. , Tantama, M. & Yellen, G. (2011) Imaging cytosolic NADH‐NAD^+^ redox state with a genetically encoded fluorescent biosensor. Cell Metabolism, 14, 545–554. 21982714 10.1016/j.cmet.2011.08.012 PMC3190165

[tpj16796-bib-0013] Kasimova, M.R. , Grigiene, J. , Krab, K. , Hagedorn, P.H. , Flyvbjerg, H. , Andersen, P.E. et al. (2006) The free NADH concentration is kept constant in plant mitochondria under different metabolic conditions. Plant Cell, 18, 688–698. 16461578 10.1105/tpc.105.039354 PMC1383643

[tpj16796-bib-0014] Latouche, G. , Cerovic, Z.G. , Montagnini, F. & Moya, I. (2000) Light‐induced changes of NADPH fluorescence in isolated chloroplasts: a spectral and fluorescence lifetime study. Biochimica et Biophysica Acta, 1460, 311–329. 11106772 10.1016/s0005-2728(00)00198-5

[tpj16796-bib-0015] Lim, S.L. , Flutsch, S. , Liu, J. , Distefano, L. , Santelia, D. & Lim, B.L. (2022) Arabidopsis guard cell chloroplasts import cytosolic ATP for starch turnover and stomatal opening. Nature Communications, 13, 652. 10.1038/s41467-022-28263-2 PMC8814037 35115512

[tpj16796-bib-0016] Lim, S.L. , Voon, C.P. , Guan, X. , Yang, Y. , Gardestrom, P. & Lim, B.L. (2020) *In planta* study of photosynthesis and photorespiration using NADPH and NADH/NAD^+^ fluorescent protein sensors. Nature Communications, 11, 3238. 10.1038/s41467-020-17056-0 PMC7320160 32591540

[tpj16796-bib-0017] Liu, J. , Lim, S.L. , Zhong, J.Y. & Lim, B.L. (2022) Bioenergetics of pollen tube growth in *Arabidopsis thaliana* revealed by ratiometric genetically encoded biosensors. Nature Communications, 13, 1–19. 10.1038/s41467-022-35486-w PMC9763403 36535933

[tpj16796-bib-0018] Lowry, O.H. & Passonneau, J.V. (1972) Pyridine nucleotides. In: A flexible system of enzymatic analysis. New York: Academic Press, pp. 13–20.

[tpj16796-bib-0019] Molinari, P.E. , Krapp, A.R. , Weiner, A. , Beyer, H.M. , Kondadi, A.K. , Blomeier, T. et al. (2023) NERNST: a genetically‐encoded ratiometric non‐destructive sensing tool to estimate NADP (H) redox status in bacterial, plant and animal systems. Nature Communications, 14, 3277. 10.1038/s41467-023-38739-4 PMC10244373 37280202

[tpj16796-bib-0020] Müller‐Schüssele, S.J. , Schwarzländer, M. & Meyer, A.J. (2021) Live monitoring of plant redox and energy physiology with genetically encoded biosensors. Plant Physiology, 186, 93–109. 34623445 10.1093/plphys/kiab019 PMC8154060

[tpj16796-bib-0021] Murashige, T. & Skoog, F. (1962) A revised medium for rapid growth and bio assays with tobacco tissue cultures. Physiologia Plantarum, 15, 473–497.

[tpj16796-bib-0022] Ovečka, M. , Berson, T. , Beck, M. , Derksen, J. , Šamaj, J. , Baluška, F. et al. (2010) Structural sterols are involved in both the initiation and tip growth of root hairs in *Arabidopsis thaliana* . Plant Cell, 22, 2999–3019. 20841426 10.1105/tpc.109.069880 PMC2965552

[tpj16796-bib-0023] Rowe, J.H. & Jones, A.M. (2021) Focus on biosensors: looking through the lens of quantitative biology. Quantitative Plant Biology, 2, e12. 37077214 10.1017/qpb.2021.10 PMC10095858

[tpj16796-bib-0024] Shaner, N.C. , Campbell, R.E. , Steinbach, P.A. , Giepmans, B.N. , Palmer, A.E. & Tsien, R.Y. (2004) Improved monomeric red, orange and yellow fluorescent proteins derived from Discosoma sp. red fluorescent protein. Nature Biotechnology, 22, 1567–1572. 10.1038/nbt1037 15558047

[tpj16796-bib-0025] Shen, J.B. , Zeng, Y.L. , Zhuang, X.H. , Sun, L. , Yao, X.Q. , Pimpl, P. et al. (2013) Organelle pH in the Arabidopsis endomembrane system. Molecular Plant, 6, 1419–1437. 23702593 10.1093/mp/sst079

[tpj16796-bib-0026] Singh, G. , Pereira, D. , Baudrey, S. , Hoffmann, E. , Ryckelynck, M. , Asnacios, A. et al. (2021) Real‐time tracking of root hair nucleus morphodynamics using a microfluidic approach. The Plant Journal, 108, 303–313. 34562320 10.1111/tpj.15511

[tpj16796-bib-0027] Steinbeck, J. , Fuchs, P. , Negroni, Y.L. , Elsässer, M. , Lichtenauer, S. , Stockdreher, Y. et al. (2020) *In vivo* NADH/NAD^+^ biosensing reveals the dynamics of cytosolic redox metabolism in plants. Plant Cell, 32, 3324–3345. 32796121 10.1105/tpc.20.00241 PMC7534465

[tpj16796-bib-0028] Tao, R.K. , Zhao, Y.Z. , Chu, H.Y. , Wang, A.X. , Zhu, J.H. , Chen, X.J. et al. (2017) Genetically encoded fluorescent sensors reveal dynamic regulation of NADPH metabolism. Nature Methods, 14, 720–728. 28581494 10.1038/nmeth.4306 PMC5555402

[tpj16796-bib-0029] Voon, C.P. , Guan, X. , Sun, Y. , Sahu, A. , Chan, M.N. , Gardeström, P. et al. (2018) ATP compartmentation in plastids and cytosol of *Arabidopsis thaliana* revealed by fluorescent protein sensing. Proceedings of the National Academy of Sciences of the United States of America, 115, 10778–10787. 10.1073/pnas.1711497115 PMC6233094 30352850

[tpj16796-bib-0030] Wagner, S. , Nietzel, T. , Aller, I. , Costa, A. , Fricker, M.D. , Meyer, A.J. et al. (2015) Analysis of plant mitochondrial function using fluorescent protein sensors. Methods in Molecular Biology, 1305, 241–252. 25910739 10.1007/978-1-4939-2639-8_17

[tpj16796-bib-0031] Wagner, S. , Steinbeck, J. , Fuchs, P. , Lichtenauer, S. , Elsässer, M. , Schippers, J.H. et al. (2019) Multiparametric real‐time sensing of cytosolic physiology links hypoxia responses to mitochondrial electron transport. New Phytologist, 224, 1668–1684. 31386759 10.1111/nph.16093

[tpj16796-bib-0032] Zhao, Y.Z. , Hu, Q.X. , Cheng, F.X. , Su, N. , Wang, A.X. , Zou, Y.J. et al. (2015) SoNar, a highly responsive NAD^+^/NADH sensor, allows high‐throughput metabolic screening of anti‐tumor agents. Cell Metabolism, 21, 777–789. 25955212 10.1016/j.cmet.2015.04.009 PMC4427571

