# Supplementary material for: In planta imaging of pyridine nucleotides using second‐generation fluorescent protein biosensors
Source: Plant J. 2024 May 18;119(3):1643–58. doi: 10.1111/tpj.16796 (PMC13087476; doi:10.1111/tpj.16796)
Supplement: Supplementary file 11 — Figure S1. mC‐SoNar and mC‐iNAPs expression in Arabidopsis pollen tubes under the control of LAT52 promoter. Figure S2. Chloroplasts in the pavement cell. Figure S3. Emission spectra of 21‐day‐old plants expressing different biosensors. Figure S4. An overview of the confocal and ratio images of two independent lines for each of the biosensors in 5‐day‐old seedlings. Table S1. Summary of biosensors used in Arabidopsis research. Table S2. Details of genetically encoded pyridine nucleotide biosensor transgenic Arabidopsis lines deposited at ABRC. Table S3. Primer sequences used for plasmid constructions. [file TPJ-119-1643-s004.docx]

**Supporting Information**

# Article title: *In planta* imaging of pyridine nucleotides using second-generation fluorescent protein biosensors

# Authors: Shey-Li Lim, Jinhong Liu, Gilles Dupouy, Gaurav Singh, Stéphanie Baudrey, Lang Yang, Jia Yi Zhong, Marie-Edith Chabouté, Boon Leong Lim

**The following Supporting Information is available for this article:**

**Figure S1.** mC-SoNar and mC-iNAPs expression in Arabidopsis pollen tubes under the control of LAT52 promoter.

**Figure S2.** Chloroplasts in the pavement cell.

**Figure S3.** Emission spectra of 21-day-old plants expressing different biosensors.

**Figure S4.** An overview of the confocal and ratio images of two independent lines for each of the biosensors in 5-day-old seedlings.

**Table S1.** Summary of biosensors used in Arabidopsis research.

**Table S2.** Details of genetically encoded pyridine nucleotide biosensor transgenic Arabidopsis lines deposited at ABRC.

**Table S3**. Primer sequences used for plasmid constructions.

**We also provide a separate Supplemental Materials and Methods file for this article, which include the following tables:**

**Table S4:** Excitation and emission wavelengths setup for confocal microscopy.

**Table S5:** Custom MATLAB probe parameters.

**Table S6:** Troubleshooting table.

**
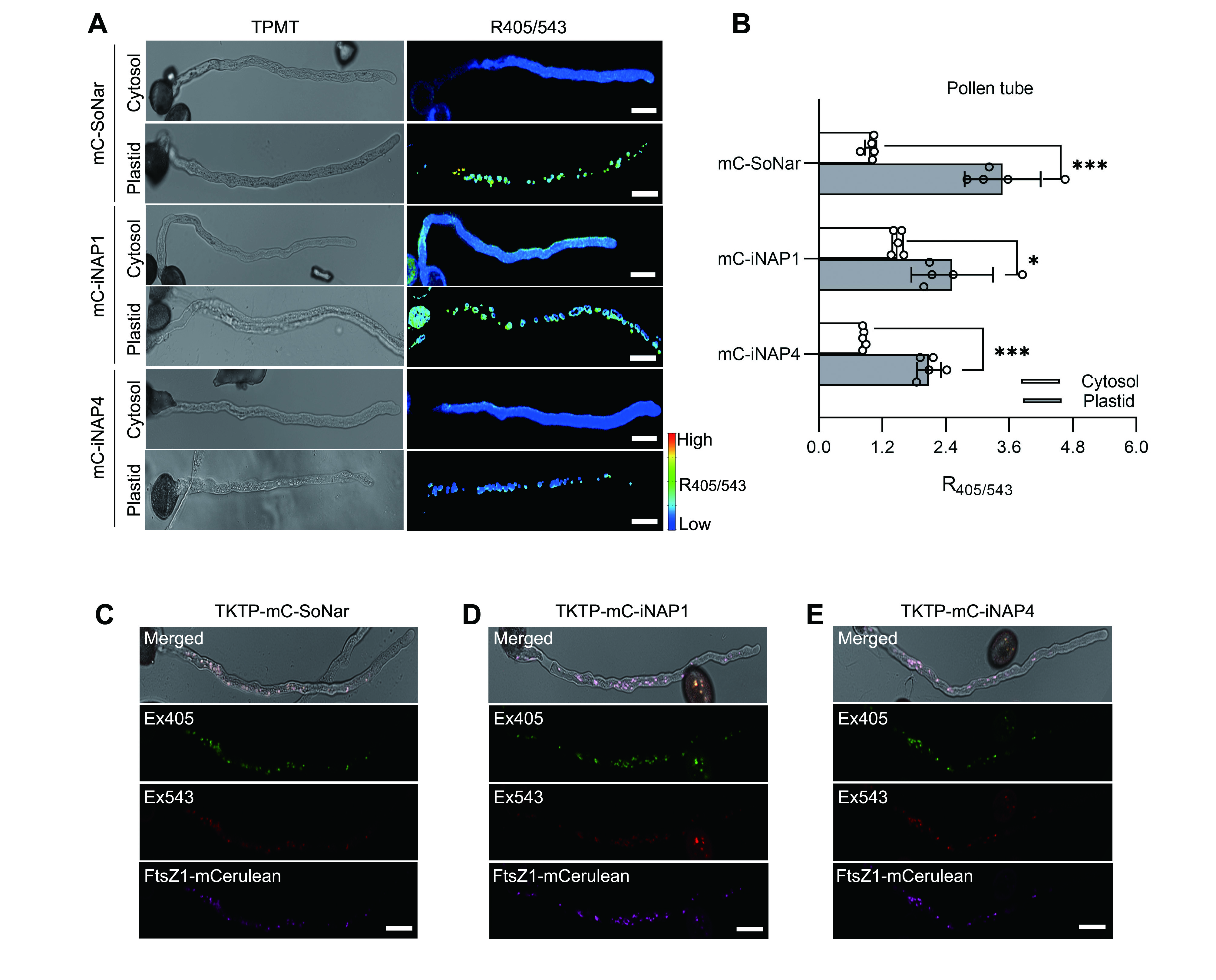
**

**Figure S1**. **mC-SoNar and mC-iNAPs expression in Arabidopsis pollen tubes under the control of LAT52 promoter.**

**(A)** Ratio images of mC-SoNar and mC-iNAPs expressed in pollen tube cytosol or plastids (TKTP) using the confocal microscope. White scale bar, 20 µm. **(B)** Comparison of the ratios of mC-SoNar or mC-iNAPs between the cytosol and plastids of pollen tubes (unpaired t-tests, two-tailed at *P < 0.05; ***P < 0.001; *n* =5; mean ± SD). Pollen tubes from the crossing lines of **(C)** mC-SoNar, **(D)** mC-iNAP1, and **(E)** mC-iNAP4 with FtsZ1-mCerulean were observed and their co-localization in plastid pollen was verified. Biosensor signals were excited at 405 nm (green) and 543 nm (red), and emissions were detected at 545 ± 20 nm and 630 ± 60 nm, respectively, while mCerulean was excited at 458 nm with the emission of 478 ± 13 nm (violet). Scale bars, 20 µm; mC, mCherry.


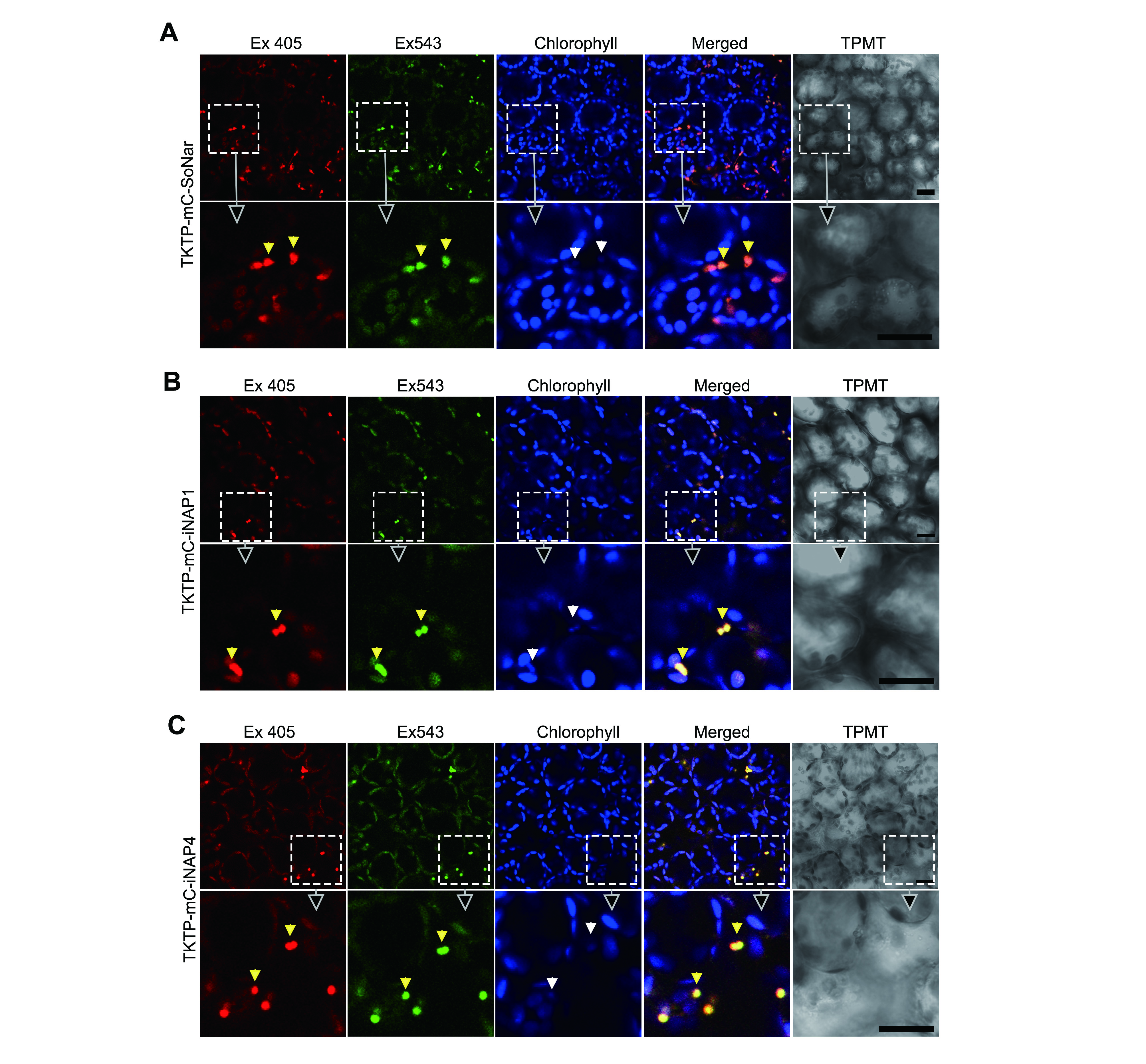
**Figure S2. Chloroplasts in the pavement cell.**

Chloroplasts in the pavement cells and mesophyll cells of 5-day-old seedlings of **(A)** mC-SoNar, **(B)** mC-iNAP1, and **(C)** mC-iNAP4 were observed and their co-localization with auto chlorophyll fluorescence. Biosensor signals were excited at 405 nm (red) and 543 nm (green), and emissions were detected at 520 ± 16 nm and 609 ± 25 nm, respectively. The subsets of the specific blot images in the white dashed boxes are magnified. White arrowheads, auto chlorophyll fluorescence. Yellow arrowheads, biosensor signals from chloroplasts in the pavement cell. Black scale bars, 20 µm**;** mC, mCherry.

**
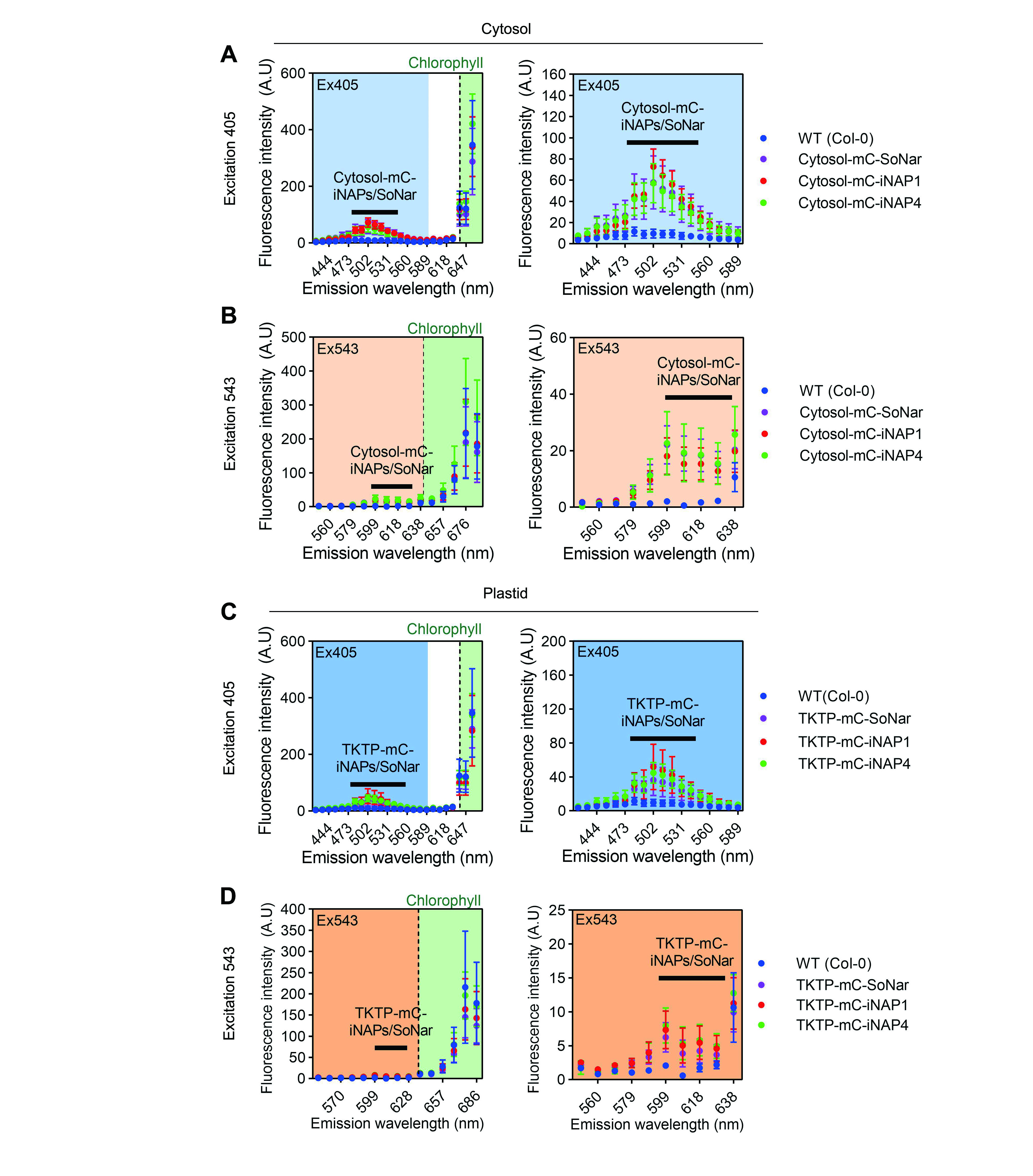
**

**Figure S3**. **Emission spectra of 21-day-old plants expressing different biosensors**.

Emission wavelengths of 3-week-old plants expressing no biosensor (WT Col-0), or mC-SoNar, mC-iNAP1, and mC-iNAP4 biosensors in, **(A-B)** cytosol, **(C-D)** Plastid stroma (TKTP) were recorded with excitation wavelengths at 405 nm (blue graphs) or 543 nm (orange graphs). The emission was collected with a resolution of 9.6 nm (*n* >15 plants; mean ± SD). The subset of the specific emission wavelengths between 434 nm to 589 nm, and 550 nm to 638 nm were presented in the enlarged colour patch plot. Black bars indicate the acquisition range of biosensor emission wavelengths throughout the study. The green area indicates autofluorescence of chlorophyll. A.U., arbitrary units; mC, mCherry.


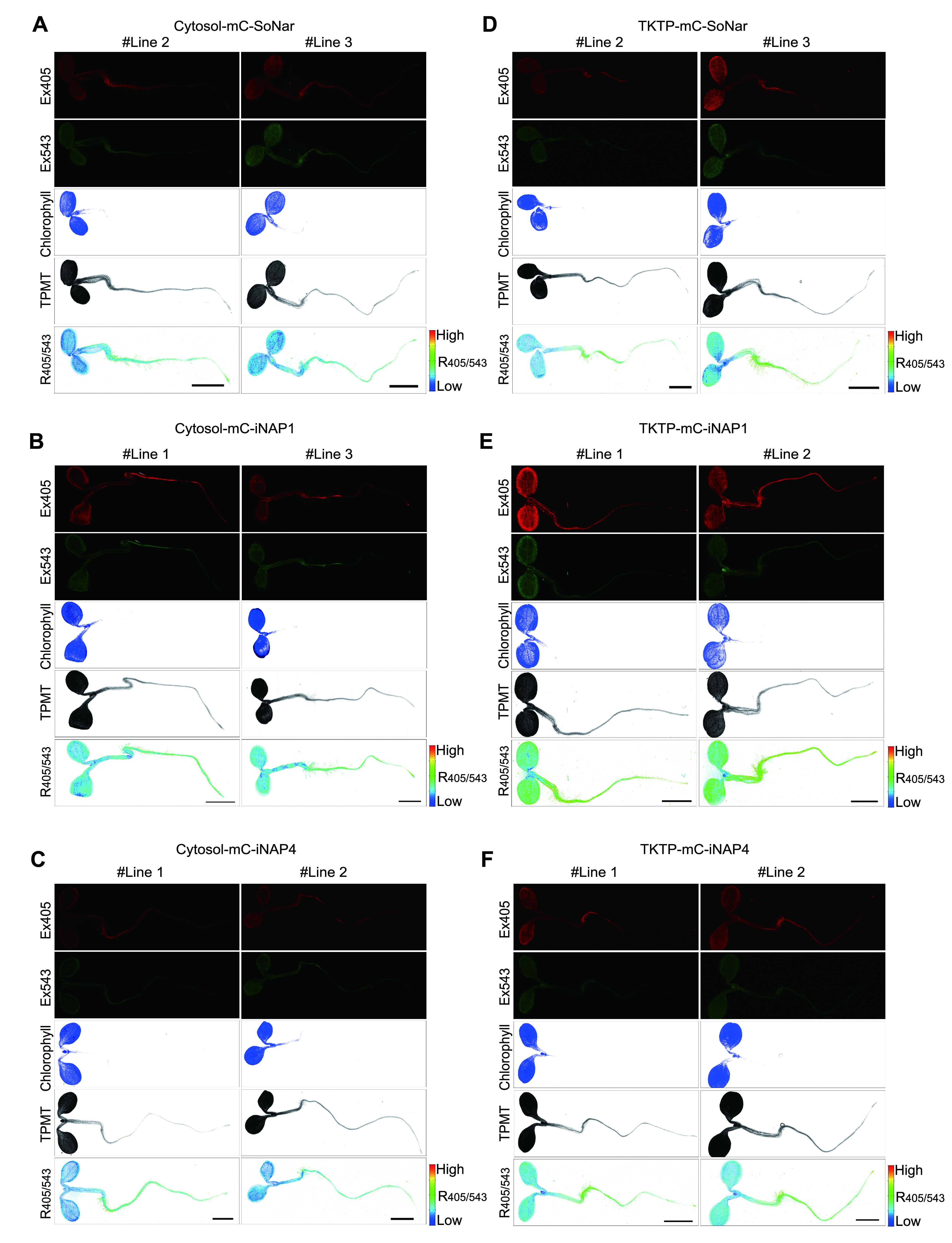


**Figure S4. An overview of the confocal and ratio images of two independent lines for each of the biosensors in 5-day-old seedlings.**

**(A)** Cytosolic mC-SoNar #2 and #3, **(B)** Cytosolic mC-iNAP1 #1 and #3 , **(C)** Cytosolic mC-iNAP4 #1 and #2, **(D)** Plastid (TKTP) mC-SoNar #2 and #3, **(E)** Plastid mC-iNAP1 #1 and #2, and **(F)** Plastid mC-iNAP4 #1 and #2. The images were acquired using confocal microscope tile scan mode with dual excitation at 405 nm and 543 nm, and the emissions were set at 520 ± 16 nm and 609 ± 25 nm respectively. The chlorophyll was detected at excitation of 488 nm, and emission of 610 nm. Ratio images are presented in pseudocolor where high R405/543 ratios (red) correspond to high NADH/NAD^+^ ratios or NADPH levels. Scale bars, 2000 µm; mC, mCherry.

**Table S1. Summary of biosensors used in Arabidopsis research.**

| **Biosensor** | **Target** | **Type of fluorescent protein** | **References** |
| --- | --- | --- | --- |
| H148D | pH | Ratiometric (GFP) | (Fasano *et al.*, 2001) |
| pHusion | pH | Ratiometric (mRFP1, EGFP) | (Lorenzen I *et al.*, 2004) |
| cpYFP | pH | Ratiometric (cpYFP) | (Schwarzlander *et al.*, 2011, Voon *et al.*, 2018, Lim *et al.*, 2022) |
| PRpHluorin | pH | Ratiometric (GFP) | (Shen *et al.*, 2013) |
| YC2.1 | Ca^2+^ | FRET (ECFP, EYFP) | (Allen *et al.*, 1999) |
| YC3.1 | Ca^2+^ | FRET (ECFP, EYFP) | (Iwano *et al.*, 2004) |
| YC3.6 | Ca^2+^ | FRET (ECFP, cp173Venus) | (Iwano *et al.*, 2009, De Col *et al.*, 2017) |
| YC4.6 | Ca^2+^ | FRET (ECFP, cp173Venus) | (Iwano *et al.*, 2009) |
| D3cpv | Ca^2+^ | FRET (CFP, YFP) | (Costa *et al.*, 2010) |
| R-GECO1 | Ca^2+^ | Ratiometric (cpmApple) | (Keinath *et al.*, 2015) |
| R-GECO1-mTurquoise | Ca^2+^ | Ratiometric (mTurquoise, cpmApple) | (Waadt *et al.*, 2017) |
| GCaMP3 | Ca^2+^ | Ratiometric (EGFP) | (Vincent *et al.*, 2017) |
| SoNar | NADH/NAD^+^ | Ratiometric (cpYFP) | (Lim *et al.*, 2020, Lim *et al.*, 2022) |
| Peredox-mCherry | NADH/NAD^+^ | Ratiometric(tSapphire,mCherry) | (Steinbeck *et al.*, 2020) |
| Peredox-mCherry-DS | NADH/NAD^+^ | Ratiometric (tsapphire, mCherry) | (Steinbeck *et al.*, 2020) |
| mCherry-SoNar | NADH/NAD^+^ | Ratiometric(mCherry, cpYFP) | (Liu *et al.*, 2022) |
| iNAP | NADPH | Ratiometric(cpYFP) | (Lim *et al.*, 2020, Lim *et al.*, 2022) |
| mCherry-iNAP | NADPH | Ratiometric (mCherry, cpYFP) | (Liu *et al.*, 2022) |
| NERST | NADP(H) | Ratiometric (roGFP2) | (Molinari *et al.*, 2023) |
| ATeam1.03 | MgATP^2-^ | FRET (mseCFP, mVenus) | (De Col *et al.*, 2017, Voon *et al.*, 2018, Zhang *et al.*, 2018, Lim *et al.*, 2022, Liu *et al.*, 2022) |
| NiMet3.0 | Nitrate | FRET (edAFP, edeCFP) | (Chen *et al.*, 2022) |
| Grx1-roGFP1 | Glutathione | Ratiometric (GFP) | (Jiang *et al.*, 2006) |
| Grx1-roGFP2 | Glutathione | Ratiometric (GFP) | (Meyer *et al.*, 2007) |
| Hyper | H_2_O_2_ | Ratiometric (GFP) | (Costa *et al.*, 2010) |
| roGFP-Orp1 | H_2_O_2_ | Ratiometric (GFP) | (Nietzel *et al.*, 2019) |
| Hyper7 | H_2_O_2_ | Ratiometric (GFP) | (Ugalde *et al.*, 2021) |
| roGFP2-Tsa2ΔCR | H_2_O_2_ | Ratiometric (GFP) | (Lampl *et al.*, 2022) |
| cpFLIPPi | Phosphate | FRET (eCFP, cpVenus) | (Mukherjee *et al.*, 2015, Zhang *et al.*, 2018) |
| FLIPglu-Δ13 | Glucose | FRET (eCFP, eYFP) | (Deuschle *et al.*, 2006) |
| FLIPglu–600μΔ | Glucose | FRET (eCFP, eYFP) | (Chaudhuri *et al.*, 2008) |
| FLIPsuc–90μΔ1 | Sucrose | FRET (eCFP, eYFP) | (Chaudhuri *et al.*, 2008) |
| iGluSnFR | Glutamate | Ratiometric (cpGFP) | (Toyota *et al.*, 2018) |
| ABAleon2.1 | Abscisic acid | FRET (mTurquoise, cpVenus173) | (Waadt *et al.*, 2014) |
| ABACUS1 | Abscisic acid | FRET (edCerulean, edCitrine) | (Jones *et al.*, 2014) |
| GPS1 | Gibberellin | FRET (CFP, YFP) | (Rizza *et al.*, 2017) |
| PAleon | Phosphatidic acid | FRET (CFP, Venus) | (Li *et al.*, 2019) |
| GFP–N160RbohD | Phosphatidic acid | Ratiometric (GFP) | (Li *et al.*, 2022) |
| Clomeleon | Chloride & Nitrate | FRET (CFP, YFP) | (Lorenzen I *et al.*, 2004) |
| ClopHensor | H^+^ & Chloride & Nitrate | Ratiometric (E^2GF, DsRed) | (Demes *et al.*, 2020) |

**Table S2.** **Details of genetically encoded pyridine nucleotide biosensor transgenic Arabidopsis lines deposited at ABRC.**

| **Biosensor** | **Description** | **ABRC stock number** |
| --- | --- | --- |
| **CaMV 35S second-generation pyridine nucleotide biosensor** | | |
| pH7WG2-mCherry-iNAP1 | High affinity cytosolic NADPH-based biosensor mCherry-iNAP1 expressed from the CaMV 35S promoter. This is a heterozygous third generation transformed line (T3). | CS73429 |
| pH7WG2-mCherry-iNAP4 | Low affinity cytosolic NADPH-based biosensor mCherry-iNAP4 expressed from the CaMV 35S promoter. This is a T3 homozygous line. | CS73430 |
| pH7WG2-mCherry-SoNar | Cytosolic NADH/NAD^+^-based biosensor mCherry-SoNar expressed from the CaMV 35S promoter. This is a T3 heterozygous line. | CS73431 |
| pH7WG2-TKTP-mCherry-iNAP1 | High affinity plastid stroma NADPH-based biosensor mCherry-iNAP1 expressed from the CaMV 35S promoter. This is a T3 heterozygous line. | CS73432 |
| pH7WG2-TKTP-mCherry-iNAP4 | Low affinity plastid stroma NADPH-based biosensor mCherry-iNAP4 expressed from the CaMV 35S promoter. This is a T3 homozygous line. | CS73433 |
| pH7WG2-TKTP-mCherry-SoNar | Plastid stroma NADH/NAD^+^-based biosensor mCherry-SoNar expressed from the CaMV 35S promoter. This is a T3 homozygous line. | CS73434 |
| **CaMV 35S first-generation pyridine nucleotide biosensor** | | |
| pEarley100-iNAP1 | High affinity cytosolic NADPH-based biosensor iNAP1 expressed from the CaMV 35S promoter. This is a heterozygous second generation transformed line (T2). | CS73421 |
| pEarley100-SoNar | Cytosolic NADH/NAD^+^-based biosensor SoNar expressed from the CaMV 35S promoter. This is a T2 heterozygous line. | CS73422 |
| pEarley100-iNAPc | Cytosolic pH control biosensor for cytosolic iNAP1 and SoNar. A iNAPc expressed from the CaMV 35S promoter. This is a T2 heterozygous line. | CS73423 |
| pEarley100-TKTP-iNAP4 | Low affinity plastid stroma NADPH-based biosensor iNAP4 expressed from the CaMV 35S promoter. This is a T2 heterozygous line. | CS73424 |
| pEarley100-TKTP-SoNar | Plastid stroma NADH/NAD^+^-based biosensor SoNar expressed from the CaMV 35S promoter. This is a T2 heterozygous line. | CS73425 |
| pEarley100-TKTP-iNAPc | Plastid stroma pH control biosensor for TKTP-iNAP1 and TKTP-SoNar. A iNAPc expressed from the CaMV 35S promoter. This is a T2 heterozygous line. | CS73426 |
| pEarley100-iNAP4-SRL | Peroxisome NADPH biosensor iNAP4 expressed from the CaMV 35S promoter. This is a T2 heterozygous line. | CS73427 |
| pEarley100-iNAPc-SRL | Peroxisome pH control biosensor for iNAP4-SRL. A iNAPc expressed from the CaMV 35S promoter. This is a T2 heterozygous line. | CS73428 |
| **LAT52 second-generation pyridine nucleotide biosensor** | | |
| pEarley302-LAT52-mCherry-iNAP1 | High affinity pollen cytosolic NADPH-based biosensor mCherry-iNAP1 expressed from the LAT52 promoter. This is a T3 homozygous line. | CS73409 |
| pEarley302-LAT52-mCherry-iNAP4 | Low affinity pollen cytosolic NADPH-based biosensor mCherry-iNAP4 expressed from the LAT52 promoter. This is a T3 homozygous line. | CS73410 |
| pEarley302-LAT52-mCherry-SoNar | Pollen cytosolic NADH/NAD^+^-based biosensor mCherry-SoNar expressed from the LAT52 promoter. This is a T3 homozygous line. | CS73411 |
| pEarley302-LAT52-TKTP-mCherry-iNAP1 | High affinity pollen plastid stroma NADPH-based biosensor mCherry-iNAP1 expressed from the LAT52 promoter. This is a T3 homozygous line. | CS73413 |
| pEarley302-LAT52-TKTP-mCherry-iNAP4 | Low affinity pollen plastid stroma NADPH-based biosensor mCherry-iNAP4 expressed from the LAT52 promoter. This is a T3 homozygous line. | CS73414 |
| pEarley302-LAT52-TKTP-mCherry-SoNar | Pollen plastid stroma NADH/NAD^+^-based biosensor mCherry-SoNar expressed from the LAT52 promoter. This is a T3 homozygous line. | CS73415 |

**Table S3**. **Primer sequences used for plasmid constructions.**

| **Primer** | **Sequence (5’– 3’)** | **Purpose** |
| --- | --- | --- |
| mCherry-F | TAATGAATTCATGTTGAGCAAGGGCGAGGAGGAT | Amplify mCherry cDNA  Addition of (GGSGG)_4_ (bold) |
| mCherry- R | ATTAGGATCC**ACCTCCTCCAGAACCTCCTCCTCCAGAACCTCC**CTTGTACAGCTCGTCCATGCCG |  |
| iNAP/Sonar-F | TAATGGATCC**GGAGGAGGAGGTTCTGGAGGA**ATGAACCGGAAGTGGGGCCT | Amplify iNAP/SoNar cDNA  Addition of (GGSGG)**_4_** (bold) |
| iNAP/Sonar-R | ATTAAAGCTTTTAGCCCATCATCTCCTCCCGCC |  |
| mCherry-M | CGGCGCCTACAACGTCAACATC | Sequencing |
| mCherry-iNAP/SoNar- F | AGCGCGGATCCGCGACTAGTATGTTGAGCAAGGGCGAGGA | Amplify mCherry-iNAP/SoNar cDNA |
| mCherry- iNAP/SoNar-R | CGCCCACCCTTGGGTCTAGATCAGCCCATCATCTCCTCCC |  |

**References**

**Allen, G.J., Kwak, J.M., Chu, S.P., Llopis, J., Tsien, R.Y., Harper, J.F. and Schroeder, J.I.** (1999) Cameleon calcium indicator reports cytoplasmic calcium dynamics in Arabidopsis guard cells. *The Plant Journal*, **19**, 735-747.

**Chaudhuri, B., Hormann, F., Lalonde, S., Brady, S.M., Orlando, D.A., Benfey, P. and Frommer, W.B.** (2008) Protonophore- and pH-insensitive glucose and sucrose accumulation detected by FRET nanosensors in Arabidopsis root tips. *The Plant Journal*, **56**, 948-962.

**Chen, Y.-N., Cartwright, H.N. and Ho, C.-H.** (2022) In vivo visualization of nitrate dynamics using a genetically encoded fluorescent biosensor. *Science Advances*, **8**, eabq4915.

**Costa, A., Drago, I., Behera, S., Zottini, M., Pizzo, P., Schroeder, J.I., Pozzan, T. and Schiavo, F.L.** (2010) H2O2 in plant peroxisomes: an in vivo analysis uncovers a Ca2+‐dependent scavenging system. *The Plant Journal*, **62**, 760-772.

**De Col, V., Fuchs, P., Nietzel, T., Elsässer, M., Voon, C.P., Candeo, A., Seeliger, I., Fricker, M.D., Grefen, C., Møller, I.M., Bassi, A., Lim, B.L., Zancani, M., Meyer, A.J., Costa, A., Wagner, R. and Schwarzländer, M.** (2017) ATP sensing in living plant cells reveals tissue gradients and stress dynamics of energy physiology. *eLife*, **6**, 26770.

**Demes, E., Besse, L., Cubero-Font, P., Satiat-Jeunemaitre, B., Thomine, S. and De Angeli, A.** (2020) Dynamic measurement of cytosolic pH and [NO_3_^−^] uncovers the role of the vacuolar transporter AtCLCa in cytosolic pH homeostasis. *Proceedings of the National Academy of Sciences of the United States of America*, **117**, 15343-15353.

**Deuschle, K., Chaudhuri, B., Okumoto, S., Lager, I., Lalonde, S. and Frommer, W.B.** (2006) Rapid metabolism of glucose detected with FRET glucose nanosensors in epidermal cells and intact roots of Arabidopsis RNA-silencing mutants. *Plant Cell*, **18**, 2314-2325.

**Fasano, J.M., Swanson, S.J., Blancaflor, E.B., Dowd, P.E., Kao, T.-h. and Gilroy, S.** (2001) Changes in root cap pH are required for the gravity response of the Arabidopsis root. *Plant Cell*, **13**, 907-921.

**Iwano, M., Entani, T., Shiba, H., Kakita, M., Nagai, T., Mizuno, H., Miyawaki, A., Shoji, T., Kubo, K. and Isogai, A.** (2009) Fine-tuning of the cytoplasmic Ca2+ concentration is essential for pollen tube growth. *Plant Physiol*, **150**, 1322-1334.

**Iwano, M., Shiba, H., Miwa, T., Che, F.-S., Takayama, S., Nagai, T., Miyawaki, A. and Isogai, A.** (2004) Ca2+ dynamics in a pollen grain and papilla cell during pollination of Arabidopsis. *Plant Physiol*, **136**, 3562-3571.

**Jiang, K., Schwarzer, C., Lally, E., Zhang, S., Ruzin, S., Machen, T., Remington, S.J. and Feldman, L.** (2006) Expression and characterization of a redox-sensing green fluorescent protein (reduction-oxidation-sensitive green fluorescent protein) in Arabidopsis. *Plant Physiol*, **141**, 397-403.

**Jones, A.M., Danielson, J.Å., ManojKumar, S.N., Lanquar, V., Grossmann, G. and Frommer, W.B.** (2014) Abscisic acid dynamics in roots detected with genetically encoded FRET sensors. *elife*, **3**, e01741.

**Keinath, N.F., Waadt, R., Brugman, R., Schroeder, J.I., Grossmann, G., Schumacher, K. and Krebs, M.** (2015) Live cell imaging with R-GECO1 sheds light on flg22-and chitin-induced transient [Ca2+] cyt patterns in Arabidopsis. *Mol Plant*, **8**, 1188-1200.

**Lampl, N., Lev, R., Nissan, I., Gilad, G., Hipsch, M. and Rosenwasser, S.** (2022) Systematic monitoring of 2-Cys peroxiredoxin-derived redox signals unveiled its role in attenuating carbon assimilation rate. *Proceedings of the National Academy of Sciences of the United States of America*, **119**, e2119719119.

**Li, T., Xiao, X., Liu, Q., Li, W., Li, L., Zhang, W., Munnik, T., Wang, X. and Zhang, Q.** (2022) Dynamic responses of PA to environmental stimuli imaged by a genetically encoded mobilizable fluorescent sensor. *Plant Communications*, 100500.

**Li, W., Song, T., Wallrad, L., Kudla, J., Wang, X. and Zhang, W.** (2019) Tissue-specific accumulation of pH-sensing phosphatidic acid determines plant stress tolerance. *Nat Plants*, **5**, 1012-1021.

**Lim, S.L., Flutsch, S., Liu, J., Distefano, L., Santelia, D. and Lim, B.L.** (2022) Arabidopsis guard cell chloroplasts import cytosolic ATP for starch turnover and stomatal opening. *Nature Communications*, **13**, 652.

**Lim, S.L., Voon, C.P., Guan, X., Yang, Y., Gardestrom, P. and Lim, B.L.** (2020) *In planta* study of photosynthesis and photorespiration using NADPH and NADH/NAD^+^ fluorescent protein sensors. *Nature Communications*, **11**, 3238.

**Liu, J., Lim, S.L., Zhong, J.Y. and Lim, B.L.** (2022) Bioenergetics of pollen tube growth in *Arabidopsis thaliana* revealed by ratiometric genetically encoded biosensors. *Nature Communications*, **13**, 1-19.

**Lorenzen I, Aberle T and C., P.** (2004) Salt stress‐induced chloride flux: a study using transgenic Arabidopsis expressing a fluorescent anion probe. *The Plant Journal*, **38**, 539-544.

**Meyer, A.J., Brach, T., Marty, L., Kreye, S., Rouhier, N., Jacquot, J.P. and Hell, R.** (2007) Redox‐sensitive GFP in Arabidopsis thaliana is a quantitative biosensor for the redox potential of the cellular glutathione redox buffer. *The Plant Journal*, **52**, 973-986.

**Molinari, P.E., Krapp, A.R., Weiner, A., Beyer, H.M., Kondadi, A.K., Blomeier, T., López, M., Bustos-Sanmamed, P., Tevere, E. and Weber, W.** (2023) NERNST: a genetically-encoded ratiometric non-destructive sensing tool to estimate NADP (H) redox status in bacterial, plant and animal systems. *Nat Commun*, **14**, 3277.

**Mukherjee, P., Banerjee, S., Wheeler, A., Ratliff, L.A., Irigoyen, S., Garcia, L.R., Lockless, S.W. and Versaw, W.K.** (2015) Live imaging of inorganic phosphate in plants with cellular and subcellular resolution. *Plant Physiology*, **167**, 628-638.

**Nietzel, T., Elsasser, M., Ruberti, C., Steinbeck, J., Ugalde, J.M., Fuchs, P., Wagner, S., Ostermann, L., Moseler, A., Lemke, P., Fricker, M.D., Muller-Schussele, S.J., Moerschbacher, B.M., Costa, A., Meyer, A.J. and Schwarzlander, M.** (2019) The fluorescent protein sensor roGFP2-Orp1 monitors *in vivo* H_2_O_2_ and thiol redox integration and elucidates intracellular H_2_O_2_ dynamics during elicitor-induced oxidative burst in Arabidopsis. *New Phytologist*, **221**, 1649-1664.

**Rizza, A., Walia, A., Lanquar, V., Frommer, W.B. and Jones, A.M.** (2017) In vivo gibberellin gradients visualized in rapidly elongating tissues. *Nat Plants*, **3**, 803-813.

**Schwarzlander, M., Logan, D.C., Fricker, M.D. and Sweetlove, L.J.** (2011) The circularly permuted yellow fluorescent protein cpYFP that has been used as a superoxide probe is highly responsive to pH but not superoxide in mitochondria: implications for the existence of superoxide 'flashes'. *Biochem J*, **437**, 381-387.

**Shen, J.B., Zeng, Y.L., Zhuang, X.H., Sun, L., Yao, X.Q., Pimpl, P. and Jiang, L.W.** (2013) Organelle pH in the Arabidopsis endomembrane system. *Mol Plant*, **6**, 1419-1437.

**Steinbeck, J., Fuchs, P., Negroni, Y.L., Elsässer, M., Lichtenauer, S., Stockdreher, Y., Feitosa-Araujo, E., Kroll, J.B., Niemeier, J.-O. and Humberg, C.** (2020) *In vivo* NADH/NAD^+^ biosensing reveals the dynamics of cytosolic redox metabolism in plants. *Plant Cell*, **32**, 3324-3345.

**Toyota, M., Spencer, D., Sawai-Toyota, S., Jiaqi, W., Zhang, T., Koo, A.J., Howe, G.A. and Gilroy, S.** (2018) Glutamate triggers long-distance, calcium-based plant defense signaling. *Science*, **361**, 1112-1115.

**Ugalde, J.M., Schlößer, M., Dongois, A., Martinière, A. and Meyer, A.J.** (2021) The latest HyPe(r) in plant H_2_O_2_ biosensing. *Plant Physiology*, **187**, 480-484.

**Vincent, T.R., Avramova, M., Canham, J., Higgins, P., Bilkey, N., Mugford, S.T., Pitino, M., Toyota, M., Gilroy, S. and Miller, A.J.** (2017) Interplay of plasma membrane and vacuolar ion channels, together with BAK1, elicits rapid cytosolic calcium elevations in Arabidopsis during aphid feeding. *Plant Cell*, **29**, 1460-1479.

**Voon, C.P., Guan, X., Sun, Y., Sahu, A., Chan, M.N., Gardeström, P., Wagner, S., Fuchs, P., Nietzel, T., Versaw, W.K., Schwarzländer, M. and Lim, B.L.** (2018) ATP compartmentation in plastids and cytosol of *Arabidopsis thaliana* revealed by fluorescent protein sensing. *Proceedings of the National Academy of Sciences of the United States of America*, **115**, 10778-10787.

**Waadt, R., Hitomi, K., Nishimura, N., Hitomi, C., Adams, S.R., Getzoff, E.D. and Schroeder, J.I.** (2014) FRET-based reporters for the direct visualization of abscisic acid concentration changes and distribution in Arabidopsis. *eLife*, **3**, e01739.

**Waadt, R., Krebs, M., Kudla, J. and Schumacher, K.** (2017) Multiparameter imaging of calcium and abscisic acid and high‐resolution quantitative calcium measurements using R‐GECO1‐mTurquoise in Arabidopsis. *New Phytologist*, **216**, 303-320.

**Zhang, W., Lo, I.M.C., Hu, L., Voon, C.P., Lim, B.L. and Versaw, W.K.** (2018) Environmental risks of nano zerovalent iron for arsenate remediation: Impacts on cytosolic levels of inorganic phosphate and MgATP^2–^ in *Arabidopsis thaliana*. *Environmental Science & Technology*, **52**, 4385-4392.
