# Supplementary material for: In planta imaging of pyridine nucleotides using second‐generation fluorescent protein biosensors
Source: Plant J. 2024 May 18;119(3):1643–58. doi: 10.1111/tpj.16796 (PMC13087476; doi:10.1111/tpj.16796)
Supplement: Supplementary file 12 — Table S4. Excitation and emission wavelengths setup for confocal microscopy. Table S5. Custom MATLAB probe parameters. Table S6. Troubleshooting table. [file TPJ-119-1643-s005.docx]

**Supplemental Materials and Methods**

# Article title: *In planta* imaging of pyridine nucleotides using second-generation fluorescent protein biosensors

# Authors: Shey-Li Lim, Jinhong Liu, Gilles Dupouy, Gaurav Singh, Stéphanie Baudrey, Lang Yang, Jia Yi Zhong, Marie-Edith Chabouté, Boon Leong Lim

# The following Supporting Information is available in Supplemental Materials and Methods:

**Table S4:** Excitation and emission wavelengths setup for confocal microscopy.

**Table S5:** Custom MATLAB probe parameters.

**Table S6:** Troubleshooting table.

**Biological materials**

**Plant materials**

- Arabidopsis transgenic seeds (mCherry-SoNar and mCherry-iNAPs lines generated by Agrobacterium-mediated transformation). Seeds are available from ABRC (Table S2).

**Reagents**

- Sterile Milli-Q water (Millipore Milli-Q lab water system, or equivalent quality water)
- Disinfecting Bleach (Clorox®)
- Murashige and Skoog Basal Medium (MS basal medium) (Sigma-Aldrich, cat. no. M5519)
- Sucrose (Sigma-Aldrich, cat. no. S0389)
- 2-(N-Morpholino) ethanesulfonic acid hydrate (MES hydrate) (Sigma-Aldrich, cat. no. M8250)
- Calcium nitrate 4-hydrate (Panreac, cat. no. 371231.121)
- Calcium chloride 2-hydrate (BDH, cat. no. 10070)
- Magnesium sulfate heptahydrate (Sigma-Aldrich, cat. no. M1880)
- Boric acid (Sigma-Aldrich, cat. no. B7901)
- Agarose (Biowest, cat. no. 202275)
- Phytagel (Sigma-Aldrich, cat. no. P8169)
- Dimethylsulfoxide (DMSO) (Sigma-Aldrich, cat. no. D8418)
- Potassium hydroxide (KOH) (Sigma-Aldrich, cat. no. P1767)
- Industrial grade ethanol (Uni-chem, cat. no. E145836PS)
- Peat soil, 70 L (Jiffy, cat. no. E12580)
- Glufosinate ammonium (Basta) (PlantMedia, cat. no. 30632007)
- Rotenone (Sigma-Aldrich, cat. no. R8875)
- 2-Thenoyltrifluoroacetone (TTFA) (Sigma-Aldrich, cat. no. T27006)
- Antimycin A from Streptomyces (Sigma-Aldrich, cat. no. A8674)
- Oligomycin A (Sigma-Aldrich, cat. no. 75351)
- Potassium cyanide (KCN) (Sigma-Aldrich, cat. no. 207810)
- Salicylhydroxamic acid (SHAM) (Sigma-Aldrich, cat. no. S607)
- Menadione sodium bisulfite (Sigma-Aldrich, cat. no. M5750)
- Hydrogen peroxide solution (BDH, cat. no. 101275M)
- 1H,1H,2H,2H-perfluorodecyltrichlorosilane 97% stabilized with Copper (ABCR, cat. no. AB111155 ).
- HFE7500 buffer (Applied thermal fluids, cat. no. 7500-40LB)
- Polydimethylsiloxane (PDMS) (SylGard 184, cat. no.761036)

**Equipment**

- pH meter (Hanna Basic pH Benchtop Meter Model HI2211)
- Forceps, straight, Dumont SS (Fine Science Tools, cat. no. 11200-33)
- Forceps, straight (Oubel, cat. no. ESD-11)
- Scalpel blade, No. 24 (Swann-Morton, cat no. 0111)
- Petri dish, 90 mm (Thermo Fisher, cat. no. 101VR20)
- Glass bottom cell culture dish, 20 mm (NEST, cat. no. 052021EQ01)
- Schott bottle, 100 mL (Duran, cat. no. 218012458)
- Microscope slide (Sail brand, cat. no. 7101)
- Cover slip 24 mm x 40 mm (BRAND®, cat. no. BR470816)
- 96 well black plate (Corning, cat no. CLS3925)
- Water puriﬁcation system (Mili-Q Direct 16 water purification system, cat. no. ZR0Q016WW)
- Water bath (Julabo Model TW8)
- Wrapping film (Parafilm M, cat. no. PM996)
- Vinyl electrical tape (3M, cat no. 1710)
- Microcentrifuge tube, 1.5 mL (BCH, cat no. 14880)
- Pipette tip, 1000 µL (Labcon, cat. no. 10418000009)
- Pipette tip, 100 µL (BCH, cat. no. 15730)
- Pipette tip, 10 µL (Geneplastix, cat. no. 221BC10)
- Micropipette 1000 µL, 200 µL, and 10 µL (Eppendorf, cat. no. 3124000121, 3123000055, and 3123000020)
- Vacuum desiccator (Bel-Art, cat. no. 420250000)
- Vacuum pump (Bio-Rad, cat no. 262 BR 2528)
- Transfer pipet (Fisherbrand, cat no. 13-711-9A)
- Cytation 1 filter cube (Bio-Tek, cat. no.7082242, 7082267, 7082244, and 7082265)
- Microwave (Panasonic Model NNST65JM)
- 10x objective, for Zeiss LSM710 confocal microscopy, 0.30 numerical aperture, working distance (WD) 5.2 mm (Zeiss, cat. no. 440330-9902-000)
- 40x objective, for Zeiss LSM710 confocal microscopy, 1.30 numerical aperture, free working distance (FWD) 0.21mm (Zeiss, cat. no. 420462-9900-000)
- Plant growth chamber (Panasonic Model MLR352)
- Tall Petri dishes 93 x 21 mm (Dutcher, cat. no. 664161)
- Cover slip 22 mm x 32 mm (Dutcher, cat. no. 100034N)
- Kimtech wipes (Kimberly Clark, cat. no. 7624)
- 1 mm biopsy punch (Harris UniCore, Cytiva, Marlborough-UK, cat. no. Z708801)
- 6 mm biopsy punch (Harris UniCore, cat. no. Z747432)
- 0.65 mL microcentrifuge tube (Ugap/Dutcher, cat. no. 015160A)
- Oxygen plasma (Diener Model Femto)
- Electric pump ( Chemyx Model Fusion 200)
- 50 mL Syringes (Terumo, cat. no. SS+50L1)
- Agani Needles (Terumo, cat. no. AN*2238R1)
- PTFE Tubing 0.56mm ID x 1.07mm OD (Adtech, cat. no. 11929445)

**Reagent setup**

**Full-strength Murashige and Skoog agar (pH 5.7-5.8)**

For 1 L full-strength Murashige and Skoog agar, add 4.4 g of MS basal medium, 20 g of sucrose, 1.0 g of MES hydrate, and 2.5 g of phytagel to 500 mL of Mili-Q water. Adjust the pH to 5.7-5.8 using KOH and fill the total volume to 1 L using Milli-Q water. Pour the autoclaved agar onto the petri dish plate and seal the plate with wrapping film after the agar has solidified. Store at 4°C until use, with a recommended maximum storage period of 1 month.

**Half-strength Murashige and Skoog buffer (pH 5.7-5.8)**

For 100 mL half-strength Murashige and Skoog buffer (1/2 MS buffer), add 0.22 g of MS basal medium, 1 g of sucrose, and 0.05 g of MES hydrate to 50 mL of Mili-Q water. Adjust the pH to 5.7-5.8 using KOH and fill the total volume to 100 mL using Milli-Q water. Prepare fresh 1/2 MS buffer each time.

**10x pollen germination master stock (pH 7.5)**

Prepare individual stock solutions of 100 mM calcium nitrate 4-hydrate, 100 mM calcium chloride 2-hydrate, 100 mM magnesium sulfate heptahydrate, and 1% (w/v) boric acid in Mili-Q water, respectively. The individual stock can be stored at -20°C with recommended maximum storage of 6 months. For a 10 mL of 10 x pollen germination master stock, add 5 mL of Mili-Q water with 1 mL calcium nitrate 4-hydrate, 1 mL calcium chloride 2-hydrate, 1 mL magnesium sulfate heptahydrate, and 1 mL of 1% (w/v) boric acid from the first prepared individual stock. Adjust the pH to 7.5 using KOH and fill the total volume to 10 mL using Milli-Q water. The 10x pollen germination master stock can be stored at 4°C until use, with recommended maximum storage of 1 month.

**Pollen germination agar**

Freshly prepare the working pollen germination agar for use. The working pollen germination agar contains 1 mM calcium nitrate 4-hydrate, 1 mM calcium chloride 2-hydrate, 1 mM magnesium sulfate heptahydrate, and 0.01 % (w/v) boric acid. It can be prepared from the 10 x pollen germination master stock. To prepare 20 mL pollen germination agar, add 2 mL of 10x pollen germination master stock, 18 % (w/v) sucrose, and 15.8 mL Mili-Q water to bring the total volume to 20 mL. Stir the medium until completely dissolved before adding 0.8% (w/v) agarose. Heat the medium in a sealed 100 mL Schott bottle using a microwave with a microwave power setting of 1,000 watts. Also, heat and shake the medium in the order of 20 s - hand shake - 10 s - hand shake - 10 s. Thereafter, allow the medium to cool for 10 min at room temperature. Pour the medium onto the 90 mm petri dish to solidify.

**Seed sterilization buffer**

Mix 10 mL of Clorox with 40 mL sterile Milli-Q to obtain a concentration of 20 % (v/v) Clorox seed disinfection buffer. Store at room temperature and cover to avoid light until use, with a maximum storage period of 2 weeks.

**1000x Basta stock**

Mix 5 mg of Basta with 1000 µL sterile Milli-Q to obtain a concentration of 25 mM. Store at -20°C until use, with a maximum storage period of 12 months.

**1000x Rotenone stock**

Mix 19.7 mg of rotenone with 1000 µL DMSO to obtain a concentration of 50 mM and cover to avoid light. Store at -20°C until use, with a maximum storage period of 12 months.

**1000x TTFA stock**

Add 22 mg of TTFA to 1000 µL DMSO to obtain a concentration of 0.1 M and cover to avoid light. Store at -20°C until use, with a maximum storage period of 12 months.

**1000x Antimycin A stock**

Add 5.4 mg of antimycin A in 1000 µL DMSO to give a concentration of 10 mM. Store at -20°C until use, with a maximum storage period of 12 months.

**1000x Oligomycin A stock**

Add 5 mg of oligomycin A to 632 µL DMSO to obtain a concentration of 10 mM. Store at -20°C until use, with a maximum storage period of 12 months.

**1000x KCN stock**

Add 32 mg of KCN to 1000 µL sterile Mili-Q water to obtain a concentration of 0.5 M and cover to avoid light. Store at -20°C until use, with a maximum storage period of 12 months.

**1000x SHAM stock**

Add 30.6 mg of SHAM to 100 µL DMSO to obtain a concentration of 2 M. Store at -20°C until use, with a maximum storage period of 12 months.

**100x Menadione stock**

Add 8.3 mg of menadione sodium bisulfite to 1000 µL sterile Mili-Q water to obtain a concentration of 30 M and cover to avoid light. Store at -20°C until use, with a maximum storage period of 6 months.

**100x H_2_O_2_ stock**

Add 102 µL H_2_O_2_ to 898 µL sterile Mili-Q water to obtain a concentration of 1 M and cover to avoid light. Store at -20°C until use, with a maximum storage period of a week.

**Procedure**

**Plant sample preparation of seedlings, pollen tube, and root hair**

Plants do not grow well in MS agar containing hygromycin, it causes short root. Screening can be done using normal full-strength MS agar and selecting the biosensor expressing seedlings under fluorescence stereomicroscope.

**Seedling cultivation:** **Timing** **1 h (seed germination), 2 d (seed stratification), 5 d, 10 d, 21 d or 45 d (seedling and plant growth)**

1. As described in Materials, reagent setup, prepare full-strength Murashige and Skoog (MS) agar plate for seeds with biosensors expressed under the control of 35S promoter. Use full-strength MS agar plate containing 25 µM Basta for seeds with biosensors expressed in pollen under the control of the LAT52 promoter.
2. Aliquot 0.1 mL transgenic seeds into 1.5 mL microcentrifuge tube.
3. Add 1 mL seed sterilization buffer and vortex for 15 min.
4. Spray 70% (v/v) ethanol onto the outer surface of the tube seed, agar plate, pipette tip box, and bottle with sterilized Milli-Q water and bring them inside the culture hood.
5. Drain off the seed sterilization buffer.
6. Wash seeds with 1 mL of sterile Milli-Q water, pipette up and down, and drain.
7. Repeat step 6 twice.
8. Pipette 1 mL of sterilized Milli-Q water on the MS agar plate.
9. Transfer the washed seeds to the MS agar plate and distribute the seeds evenly on the plate.
10. Remove the leftover water using micropipette.
11. Seal the plate using wrapping film.
12. Store and stratify the plate in the dark at 4° C for two days.
13. Transfer the plate to culture room with the photoperiod of 16:8 light: dark, 22 ° C, and culture for 5 days or 10 days according to the purpose of the experiment.
14. For seedlings expressing biosensors in pollen, transplant the Basta-resistant 10-d-old seedlings into peat soil.
15. For seedlings expressing biosensors under the control of the 35S promoter, select the fluorescent seedlings using a stereo fluorescence microscope.
    1. Place agar plates on the stereo fluorescence microscope.
    2. Adjust the core focus of the stereo fluorescence microscope until a clear field of view is revealed.
    3. Mark the fluorescence seedlings.
16. Transplant the 10-d-old fluorescence seedlings into peat soil.
17. Keep the transplanted seedlings in plant growth chamber with a photoperiod of 16:8 until further experiments (e.g., 45-d-old plants for pollen tube experiment and 21-d-old for true leave experiment).

**Pollen germination: Timing 1 h (Pollen harvest), 2-3 h (Pollen germination)**

1. Prepare pollen germination agar as described in Materials.
2. Use a marker to draw approximately (~1 cm^2^) partitions beneath the agar plate.
3. Pick the peduncle of freshly open flower at stage 13 from 45-d-old plants with forceps.
4. Invert the peduncle and touch the stigma on the agar surface.
5. Repeat step 20-21 and pick up to 4 peduncles for a single agar.
6. Seal the plate using wrapping film.
7. Place the plate into water bath at 28 °C for 2-3 h and cover the plate to avoid direct light.
8. Repeat step 15 to screen for the fluorescence pollen tubes using stereo fluorescence microscope or directly proceed to the subsequent experiment.

**Inhibitor treatment of transgenic biosensor lines for confocal imaging:** **Timing 2 h**

1. Prepare chemical stock solution as described in Materials, reagent setup.
2. Pipette 999 µL half-strength MS medium into a 1.5 mL microcentrifuge tube for the 1000x chemical stock solution or 990 µL half-strength MS medium for the 100x chemical stock solution.
3. Place 10-d-old seedlings into a microcentrifuge tube.
4. Pipette 1 µL of 1000x chemical stock solution or 10 µL of 100x chemical stock solution into a microcentrifuge tube.
5. Place the filled microcentrifuge tube on the tube rack and transfer onto the perforated plate in a vacuum desiccator.
6. Infiltrate the seedlings for 5 min using vacuum pump.
7. Slowly open the stop cock of the desiccator, a hissing sound can be heard as the air escapes.
8. Unless otherwise stated, all chemically treated samples required dark adaptation for an hour using fully covered non-transparent boxes. Proceed to step 42 for imaging.

**Seedling cultivation and CMD preparation for root hair imaging: Timing** **1 h tip preparation, 5 d seedling growth, 2 d CMD preparation, 2 h microfluidics system preparation, 4-5 d seedling growth into the CMD device.**

1. Seedling preparation in tips.
   1. Under a laminar hood, cast 25 mL of melted [½ MS; 0.5% Sucrose; 1% Agar; pH 5.7] solution into a tall petri dish.
   2. Extract 12 µL of the solution using a 200 µL pipetting tip before the agar solidifies, wait a few seconds for solidification to begin, and then place it on a sterile petri dish. When the agar has solidified, cut the pipetting tip at the 10 µL mark using a scalpel, and set it upright inside the ½ MS-filled tall petri dish using tweezers.
   3. Deposit a surface sterilized seed expressing the biosensors under the control of a 35S promoter on top of the agar surface inside the tip, close the petri dish and seal it with plant tape.
   4. After stratifying for 2 days as in step 12, allow the seed to germinate for 4 - 5 days in a growth cabinet under long-day conditions until the root tip reaches the pipette tip's edge.
2. CMD preparation
   1. From glass wafers prepared in a white room as specified by Singh, et al. ^52^ and attached inside a petri dish, weight enough liquid PDMS so that the resulting amount covers wafer with a 5 mm layer. Use a disposable cup as PDMS sicks to any surface and cannot be cleaned properly except with 70% ethanol.
   2. Add 1/10 of the PDMS weight as curing agent and mix thoroughly with a disposable fork.
   3. Pour the resulting solution onto the wafer and place the petri dish inside a vacuum cabinet to remove the air bubbles.
   4. Leave the petri dish inside a 65°C oven for at least 2 h so that the PDMS mixture solidifies.
   5. Remove the petri dish from the oven and carefully cut around the wafer pattern with a clean scalpel (use tape to remove any PDMS debris).
   6. When the PDMS starts detaching from the wafer, use the scalpel as a lever to help it detach completely.
   7. Use tape to remove any PDMS debris on the printed PDMS and clean its surface. Leave a piece of tape to protect the printed surface from dust.
   8. Clean the wafer delicately using compressed air for it to be reusable.
   9. Punch all the inlet, outlet, and plant holes using a 1 mm biopsy punch.
   10. Re-cut the sides of the PDMS block with a scalpel but not closer than 0.2 cm from the inlet and outlet holes to avoid leakage after CMD binding.
   11. Clean a glass coverslip with deionized water followed by Kimtech wipes.
   12. Wet the coverslip using deionized water again and dry it completely using compressed air.
   13. Pre-run the plasma before binding: switch on the plasma, then the pump, and wait for 2 min to allow the vacuum to decrease to 0.1 mbar.
   14. Set the power to 2.5, switch on the gas, and wait for the gas scale to reach 0.48 mbar, then do a run for 30 s.
   15. Close the gas, switch off the pump, switch the ventilation, and carefully open the door.
   16. Dispose the PDMS design face up and the glass slide on the platter inside the chamber, close the door, switch the ventilation off, then the pump on, and run again as in step 35 (m).
   17. As soon as the activation is done, open the plasma as in step 35 (n), remove the glass slide, and place the PDMS design facing down on it to bind them together. Roll a pen on top of the PDMS to make sure the binding is even.
   18. Place the chip in a 65°C oven for 15 min to finish the binding and switch the plasma off.
   19. Transfer some silane solution (1H,1H,2H,2H-Perfluorodecyltrichlorosilane 1% in HFE7500 buffer) inside a 1ml syringe connected to PTFE tubing, cut the end of the tubing at 45°, insert it inside the inlet hole and inject the solution in the CMD.
   20. Blow the solution out using compressed air through the inlet and outlet holes. The CMD is ready to use.
3. Montage of the CMD based microfluidics system for root hair.
   1. Plunge the CMD inside a 70% ethanol solution in a petri dish, and place it inside a vacuum cabinet for 5 min.
   2. Under a laminar flow hood, prepare three tubing pieces of different lengths for the input and output of the CMD: 20 cm, 10 cm, and 7cm.
   3. Prepare a tall petri dish for the CMD by pushing two holes through the lid 2.5 cm apart using a needle white-heated at a flame.
   4. Prepare a small cylinder of PDMS using the 6 mm biopsy punch, then use the 1 mm biopsy punch to punch two holes into it.
   5. Use a pair of tweezers to insert the 20 cm tubing piece into one of the holes, and the 10 cm one in the other hole.
   6. Pull both pieces of tubing through the other side of the PDMS cylinder using a pair of tweezers and cut them both at 0.5 cm from the PDMS using a scalpel.
   7. Insert the resulting PDMS cylinder inside a 0.65 mL sterile microcentrifuge tube, the cut parts of the tubing pieces facing the inside of the tube.
   8. Suck up autoclaved [½ MS; 0.5 % Sucrose; pH 5.7] liquid media inside a 50 mL syringe, add a needle at the tip, and mount both on the electrical pump.
   9. Using tweezers, delicately push the other end of the 20 cm tubing piece onto the needle.
   10. Setup the liquid flow at 2000 µL min^-1^ until it has gone through all tubing. Be careful to remove any bubbles from the system and stop the flow.
   11. Use tweezers to handle the end of the 10 cm tubing piece, cut it at a 45° angle with a sterilized scalpel, insert it through the holes from the petri dish lid, and then inside the inlet hole of the CMD.
   12. Still using tweezers, cut the remaining tubing piece the same way and insert it inside the outlet hole of the CMD.
   13. Resume the flow of liquid at 2000 µL min^-1^ until it has reached the end of the system and let it flow for 1 min to remove the remains of 70 % ethanol inside the system.

**Sample mounting: Timing 5 min (seedling sample), 5 min (pollen tube sample), 5 min (root hair sample)**

1. Sample mounting for seedling
   1. For seedling sample, prepare the imaging chamber as described in Materials, Equipment setup, confocal microscope.
   2. Put a drop of half-strength MS medium on the microscope slide.
   3. Place the seedling on the microscope slide.
   4. Cover the sample with cover slip and fix the slip with vinyl electrical tape.
2. Sample mounting for pollen tube
3. Cut the germinated pollen tube agar to 1 cm^2^ using a surgical blade.
4. Lift the agar using a 24 mm cover slip.
5. Flip the agar into the glass bottom cell culture dish.
6. The sample is ready for imaging using selected fluorescent equipment.

1. Sample mounting for root hair
   1. Set the flow rate in the CMD at 8 µL min^-1^.
   2. Extract the tip holding the seedling from the agar plate using a pair of tweezers (use a large pair for a better grip on the tip) and delicately push it through the hole until the bottom.
   3. Transfer the CMD inside the tall petri dish prepared in step 36 (c) with wet tissue paper around to keep the moisture inside the system.
   4. Put the outlet tubing through the other hole of the petri dish lid, close the dish, and seal it with parafilm.
   5. Transfer the system in a growth cabinet under the light/dark regime displayed above, increase the flow rate to 350 µL min^-1^ for 30 s while flicking the outlet tubing to remove any air bubbles inside the microchannels, and finally reduce it back to 8 µL min^-1^.
   6. Once the root has reached at least 0.5 cm inside the central channel after 3-4 days, the system can be taken out of the growth cabinet for imaging under a spinning disc microscope. Open the lid of the petri dish and break it apart to release the tubing, then place the CMD coverslip face down on the platter, remove any air bubbles from inside the channels like step 39 (e), and start imaging.
2. Select an appropriate imaging equipment for your experiments.

**High-resolution spatial imaging of pyridine nucleotides in whole plants and various tissues: Timing 5 min for lambda scan, 5 min for normal imaging, and 15 min for tile scan per seedling**

1. Lambda mode scan for mesophyll cell
   1. Mount three to four seedlings on the imaging chamber as described in step 37.
   2. Select lambda mode scan in the confocal setting and set the emission profile collection range at 9.2 nm.
   3. Select the excitation wavelength at 405 nm.
   4. Place the mounted sample on the scanning stage and start the scan.
   5. Scan the same sample for another excitation wavelength (e.g., 543 nm).
2. Normal mode scan for various tissues
3. Mount the plant on the imaging chamber as described in step 37.
4. Setup the confocal setting with 4 channels as described in Table S4.
5. Place the mounted sample on the scanning stage, find the focus, and start the scan.
6. Tile scan setting for whole plant imaging
7. Mount the plant on the imaging chamber as described in step 37.
8. Select tile scan mode in confocal setting and set the frame size and tile region accordingly. 5-d-old seedling can be acquired in the frame size of 21 mm x 8 mm.
9. Place the mounted sample on the scanning stage and start the tile scan.

**Imaging of real-time dynamic changes of pyridine nucleotides in pollen tube and root hair plastids using spinning disc microscopy:** **Timing 5 min per pollen tube, 5 min per root hair.**

1. Monitor pollen tube growth using a spinning disc microscope.
2. Germinate the pollen tube as described in step 18 – 24.
3. Mount the plant on the glass bottom cell culture dish as described in step 38.
4. Place the mounted sample on the scanning stage and start the scan.
5. Monitor root hair growth using a spinning disc microscope.
6. Prepare the CMD system and seedlings as described in steps 34-36.
7. Mount the seedlings inside the CMD system as described in step 39.
8. Once the plant root has reached at least a length of 0.5 cm inside the main channel after 4-5 days, break the petri dish apart and place the mounted sample on the scanning stage to start the scan.

**Simultaneous real-time measurement of plant tissues using multi-well fluorimetry plate reader: Timing 4 h to completely fill the 96-well plate**

1. Inhibitor study using 96-well plate.
2. Prepare 10-d-old seedlings as described in step 1-13.
3. Pipette 199.8 µL half-strength MS medium into each of the 96 well. A multichannel pipette can be used in this step.
4. Detach the shoot and root region of the seedlings using scissor.
5. Place the shoot and root into separate wells.
6. Add 0.2 µL of the 1000 x chemical solution into each well.
7. Place the filled 96-well plate on the perforated plate in a vacuum desiccator.
8. Infiltrate the seedling for 5 min using vacuum pump.
9. Slowly open the stop cock of the desiccator, a hissing sound can be heard as the air escapes.
10. Wrap the 96-well plate with aluminium foil to prevent direct light exposure.
11. Use cytation 1 fluorimetry plate reader to read the fluorescent intensities for 3h.

**Data analysis**

1. Ratio analysis using MATLAB customized software.
2. Download Version 1.3 MATLAB customized redox ratio analysis software at (<https://markfricker.org/77-2/software/redox-ratio-analysis/redox-ratio-analysis-software-download/> ).
3. Rename the RRA_12092017.mdf file to RRA_12092017.exe and install the software. MATLAB no longer ships with the Ghostscript library, download and install Ghostscript AGPL Release at ( <https://ghostscript.com/releases/gsdnld.html>).
4. Select the “Batch Process”.
5. Input the .LSM file folder into the directory of the software.
6. Create a probe based on the desired parameter as described in Table S5. Set the parameters according to the excitation wavelength setting, it is not necessary to reflect the channel input in the confocal LSM file.
7. Load the created probe database into the system and click “ok”.
8. Select “full screen” for a better viewing experience.
9. Select “Fit” in the display controls to ensure the entire image is displayed.
10. Select “Back” in the background section and double click, and drag the “cross” to select the image background area.
11. Press “Test” in the test section to obtain an overall ratio value.
12. Select “add ROI” in the set-up ROI section, if ROI selection is needed.
13. Press “Test” again in the test section to obtain the ROI ratio value.
14. Export all the images to the output directory section and press “Process” in the output section. Download Xpdf command line tools at (<https://www.xpdfreader.com/download.html>) and extract pdftops, locate pdftops file when it is instructed during data processing.
15. Ratio data is now displayed in an excel file along with the processed image.
16. Do not reanalysis the same data in the same folder. If reanalysis is required, copy the original data to a new folder prior to analysis, as the current data will be overwritten by the newly analyzed data.
17. Plastid tracking and ratio analysis using Fiji Trackmate plugin
18. Input the multi-channel time lapse into the Fiji (<https://imagej.net/software/fiji/>).
19. Draw an ROI region in the outside pollen tube area and measure the mean value of fluorescence intensity in one channel.
20. Restore the ROI selection to another channel and measure the mean fluorescence intensity also.
21. Remove the background of the two channels by subtracting the two values, respectively.
22. Call the Trackmate 7 plugin and check or set the pixel size, time interval, and total frame number, and click “Next”.
23. Select the LoG detector and set the stronger channel to be the detecting channel.
24. Set the estimated object diameter for pollen plastid to 1.6 microns. The quality threshold value depends on the video quality and tick the “Pre-process with median filter”.
25. After the preliminary spot detection, many artificial spots outside the pollen tube will be detected.
26. Set filter including quality, and mean intensity to filter out the unreliable spots.
27. Manually remove the spots outside the pollen tubes by holding the shift key and draw the unwanted ROI, then delete the background spots by using spots edit tools.
28. Select the Simple LAP tracker and set the linking/Gap-closing max distance, then the complete trajectories of each plastid will be tracked.
29. Remove the unwanted trajectories by setting filters, including the number of spots, distance, or duration in tracks et al..
30. After the plastid trajectories display, set the display option to get a clear view.
31. Get the tracking scheme, tracks, and spots information in the “Display options” panel.
32. Reorder the “Track ID” in the tracks table and export the trajectories as a .csv file for further analysis.
33. Many other data in different formats are accessed in the final action panel.
34. Read the .csv file of trajectories using Rstudio (<https://cran.r-project.org/>).
35. Extract the column of TRACK_ ID, FRAME, POSITION_T, POSITION_X, POSITION_Y, MEAN_INTENSITY_CH1, and MEAN_INTENSITY_CH2.
36. Divide the mean fluorescence intensity of two channels to get the ratio value, and remove the NA or infinite value.
37. Plot the ratio value, trajectories, and time in the stacked line chart.

**Troubleshooting**

Troubleshoot advice can be found in Table S6.

**Table S4: Excitation and emission wavelengths setup for confocal microscopy.**

| **5-d-old, 10-d-old, 21-d-old seedlings imaging setup** | | | |
| --- | --- | --- | --- |
| **Channel** | **Purpose** | **Excitation wavelength (nm)** | **Emission wavelength (nm)** |
| 1 | Autofluorescence of phenolic and lignin | 405 | 450 ± 17 |
| 2 | Fluorescent intensity of biosensor | 405 | 520 ± 16 |
| 3 | Fluorescent intensity of biosensor | 543 or 561 | 609 ± 25 |
| 4 | Autofluorescence of chlorophyll | 488 | 670 ± 30 |
| 5 | Bright field image | T-PMT | T-PMT |
| **Pollen tube imaging setup** | | | |
| **Channel** | **Purpose** | **Excitation wavelength (nm)** | **Emission wavelength (nm)** |
| 1 | Autofluorescence of phenolic and lignin | 405 | 450 ± 19 |
| 2 | Fluorescent intensity of biosensor | 405 | 539 ± 39 |
| 3 | Fluorescent intensity of biosensor | 543 or 561 | 633 ±58 |
| 4 | Bright field image | T-PMT | T-PMT |

*T-PMT, transmitted light detector

**Table S5: Custom MATLAB probe parameters.**

This probe parameter setting is based on the confocal setting from Table S4. Probe parameter settings can be adjusted accordingly for different excitation wavelength settings.

| **Input parameters** | |
| --- | --- |
| Experiment type | redox |
| Oxidised channel | 2 |
| Reduced channel | 3 |
| Autoflr channel | 1 |
| Precision | single |
| **Smoothing options** | |
| xy average | 3 |
| t average | 1 |
| Subsample | No |
| Autoflr. correction | 0.5 |
| **Ratio calculation options** | |
| SD threshold | 2 |
| Saturation limit | 0.9 |
| Calculate ratio | Normal |
| **Pseudocolour display options** | |
| Ratio scale | lin |
| Min ratio scale | 0.01 |
| Max ratio scale | 10 |
| Intensity scale | lin |
| Min intensity scale | 0 |
| Max intensity scale | 255 |
| Background colour | Black (Pollen tube)  White (5-d-old, 10-d-old, or 21-d-old seedlings) |
| **Estimated parameters** | |
| Estimated Rmin | 0.01 |
| Estimated Rmax | 10 |
| **Calibration parameters** | |
| T(k) | 298.15 |
| pH | 7 |
| Midpoint | -280 |
| I factor | 0.5 |
| Hill coefficient | 1 |

**Table S6: Troubleshooting table.**

| **Step** | **Problem** | **Possible reason** | **Solution** |
| --- | --- | --- | --- |
| 13 | Poor growth of seedlings | Uneven light exposure | Ensure plates are evenly exposed to light by placing the plate in the middle of the light source instead of the sides. |
| 13 | High contamination rate | Inadequate disinfection of seeds | Use freshly prepared sterile water each time you wash the seeds. Do not reuse the seed sanitization buffer after 2 weeks, reconstitute. |
| 14 and 16 | Low survival rate | Inappropriate acclimatization | Wrap the pot with food-wrapping plastic for at least 5 days to avoid rapid water evaporation. |
| 17 | The flowering period is too short | Plants are unhealthy and usually under stress | Proper watering, growing space, and light period. Do not let plants crowd together. |
| 24 | Poor pollen germination | Temperature is not stable, expose the sample to light | Check the water bath temperature is stable during the entire germination process and keep the plate in full dark condition |
| 31 | Seedlings become soggy after infiltration | Too high infiltration pressure | Adjust suitable vacuum infiltration pressure according to the desiccator size/volume |
| 34 | Agar drying up too quickly in the tip after sowing | The agar surface in the tip is not flat | When cutting the tip after filling with ½ MS medium, make sure to cut underneath the meniscus to leave a flat surface |
| 35 | PDMS not sticking to the coverslip | Coverslip and/or PDMS surfaces not clean | Make sure that the coverslip has been thoroughly cleaned with water and that both cover slip and PDMS surfaces have been totally dried out using compressed air |
| 41-42 | Hard to focus on the mesophyll cell | Sample surfaces are not on the same level | Gently press on the sample to make sure the seedlings appear on the same level |
| 43 | Root tile scan image is out of frame | Mounted root is not placed on the horizontal line with the cotyledons | Position the cotyledon-root perpendicular to the centre of the glass slide |
| 44 | Pollen tubes tip do not grow | Excessive exposure to fluorescent laser | Replace germinated pollen tube samples/agars more frequently to avoid reusing the same sample/agar for more than three data collection points. |
| 45 | Root hairs do not grow | Excessive temperature or contaminations | Make sure that the temperature in the growth chamber/imaging room is not exceeding 23°C and that the coverslip is not cracked |
| 46 | Inconsistent fluorescence intensity collection | Filter set could not capture the seedling fluorescence. | Place two 10-d-old seedlings in each well to ensure the wells are filled with plant samples |
